# A dynamic degradome landscape on miRNAs and their predicted targets in sugarcane caused by *Sporisorium scitamineum* stress

**DOI:** 10.1186/s12864-018-5400-8

**Published:** 2019-01-18

**Authors:** Yachun Su, Xinhuan Xiao, Hui Ling, Ning Huang, Feng Liu, Weihua Su, Yuye Zhang, Liping Xu, Khushi Muhammad, Youxiong Que

**Affiliations:** 1 Key Laboratory of Sugarcane Biology and Genetic Breeding, Ministry of Agriculture, Fujian Agriculture and Forestry University, Fuzhou, 350002 China; 2 Key Laboratory of Ministry of Education for Genetics, Breeding and Multiple Utilization of Crops, College of Crop Science, Fujian Agriculture and Forestry University, Fuzhou, 350002 China; 3 Department of Genetics, Hazara University, Mansehra, 21300 Pakistan

**Keywords:** Degradome, Sugarcane, *Sporisorium scitamineum*, Mycelial growth, Physiological index, miRNAs, miRNA target genes

## Abstract

**Background:**

Sugarcane smut is a fungal disease caused by *Sporisorium scitamineum*. Cultivation of smut-resistant sugarcane varieties is the most effective way to control this disease. The interaction between sugarcane and *S. scitamineum* is a complex network system. However, to date, there is no report on the identification of microRNA (miRNA) target genes of sugarcane in response to smut pathogen infection by degradome technology.

**Results:**

TaqMan qRT-PCR detection and enzyme activity determination showed that *S. scitamineum* rapidly proliferated and incurred significant enzyme activity changes in the reactive oxygen species metabolic pathway and phenylpropanoid metabolic pathway at 2 d and 5 d after inoculation, which was the best time points to study target gene degradation during sugarcane and *S. scitamineum* interaction. A total of 122.33 Mb of raw data was obtained from degradome sequencing analysis of YC05–179 (smut-resistant) and ROC22 (smut-susceptible) after inoculation. The Q30 of each sample was > 93%, and the sequence used for degradation site analysis exactly matched the sugarcane reference sequence. A total of 309 target genes were predicted in sugarcane, corresponding to 97 known miRNAs and 112 novel miRNAs, and 337 degradation sites, suggesting that miRNAs can efficiently direct cleavage at multiple sites in the predicted target mRNAs. Gene Ontology (GO) annotation and Kyoto Encyclopedia of Genes and Genomes (KEGG) pathway analysis indicated that the predicted target genes were involved in various regulatory processes, such as signal transduction mechanisms, inorganic ion transport and metabolism, defense mechanisms, translation, posttranslational modifications, energy production and conversion, and glycerolipid metabolism. qRT-PCR analysis of the expression level of 13 predicted target genes and their corresponding miRNAs revealed that there was no obvious negative regulatory relationship between miRNAs and their target genes. In addition, a number of putative resistance-related target genes regulated by miRNA-mediated cleavage were accumulated in sugarcane during *S. scitamineum* infection, suggesting that feedback regulation of miRNAs may be involved in the response of sugarcane to *S. scitamineum* infection.

**Conclusions:**

This study elucidates the underlying response of sugarcane to *S. scitamineum* infection, and also provides a resource for miRNAs and their predicted target genes for smut resistance improvement in sugarcane.

**Electronic supplementary material:**

The online version of this article (10.1186/s12864-018-5400-8) contains supplementary material, which is available to authorized users.

## Background

Sugarcane smut, caused by *Sporisorium scitamineum*, is a worldwide airborne fungal disease that affects sugarcane production [1]. The disease leads to severe losses in cane yield and reduces sucrose content and quality, thereby preventing further development of the sugarcane industry in China. A typical symptom of sugarcane smut involves cane tips of infected plants growing a black whip that points downwards and curls inwards around 120 d of planting. Simultaneously, mycelia invade the cane buds, and chlamydospores fall to the soil, thereby also infect sugarcane in the next growing season [2]. Compared to normal plants, the main stems of plants infected by *S. scitamineum* are small, the cane leaves are slender and light green in color, and tillers usually also grow black whips that lead to a sharp decline in sugarcane production [3]. Cultivating sugarcane varieties with excellent smut-resistance is the most economical and effective way to control the disease [4].

Due to the incomplete genome sequencing of *Saccharum* spp. hybrid, related genomics research is limited, thus hindering the progress of molecular improvement of sugarcane varieties. Current researches on the molecular mechanism of interaction between sugarcane and *S. scitamineum* mainly focus on genomics of smut pathogen [5–7], sugarcane molecular marker-assisted selection [8, 9], transcriptome [10, 11] and proteomic [12, 13] analysis, and resistance-related gene mining [10, 11]. The understanding of how miRNAs regulate the expression of their target genes in response to *S. scitamineum* infection is limited. The only earlier investigation relating to this matter was performed by our research group, which involved the identification of differentially expressed miRNAs in sugarcane challenged with *S. scitamineum* by using high-throughput sequencing [14].

MicroRNAs (miRNAs) are single-stranded, non-coding RNA molecules of approximately 21–24 nt in length in vivo [15]. miRNAs were first reported in *Caenorhabditis elegans* by Lee et al. [16]. Plant miRNAs were first obtained from a small *Arabidopsis thaliana* library by Reinhart et al. [17, 18]. miRNAs are encoded by endogenous miRNA genes and negatively regulate gene expression primarily through the degradation of target mRNAs during post-transcriptional gene silencing (PTGS) [19, 20]. miRNAs are important regulators of organisms and are commonly involved in the response of plants to biotic stress [21, 22]. Previous studies have shown that plant miRNAs enhance their resistance to pathogen infection by regulating the expression of key disease-resistance genes [22–25]. During the late stage of wheat stripe rust infection, wheat miR408 was downregulated, and the expression of target gene chemocyanin-like protein (*CLP1*) was induced, which in turn inhibited the growth of mycelia in leaf [22]. After inoculation of tomato stalks with *cucumber mosaic virus* (CMV) and *tomato aspermy virus* (TAV), miR156 was accumulated, resulting in hollow and fibrotic stalks [23]. In addition, tomato plants overexpressing miR156 showed phenotypic symptoms that are similar to that of pathogen infections, indicating that miR156 regulates the interaction of tomato with pathogens [24]. miRNAs were differentially expressed between maize varieties with high resistance and susceptibility to *Rhizoctonia solani* [25]. The expression patterns of zea-miR168a, miR-2, and miR-6 were generally upregulated in disease-resistant plants, while miR-3 was only upregulated in pathogen-infected sites [25]. The expression level of miRNA (except for miR-4 and miR-5) in *Zea mays* resistant variety was significantly higher than that in susceptible one, which induced host defense mechanisms to resist pathogen infection [25].

In organisms, the most important regulatory mechanism of miRNAs involves the miRNA-directed target cleavage that regulate their life cycle [26, 27]. Degradome sequencing, also called parallel analysis of RNA ends (PARE), is a high-throughput sequencing technique [28, 29]. This method can determine pairing information with miRNA through high-throughput deep sequencing of miRNA-mediated target gene cleavage degradation fragments, and the target genes of miRNAs are screened to determine how they regulate plant life activities in specific environments [28, 29]. Degradome sequencing has been extensively applied to the identification of miRNA target genes in crops such as wheat [28], maize [30], peanut [31], and other crops. However, due to different calculation methods and screening criteria, it is difficult to avoid false positive in degradome sequencing data. Therefore, further validation of the authenticity and reliability of the miRNAs and their target genes is needed. Previous studies have shown that qRT-PCR can effectively detect the abundance of target genes in samples and verify the reliability of sequencing results in the degradome [32]. In addition, the qRT-PCR method can also be used to detect the expression level of the corresponding miRNAs, and then verify the interaction mode between miRNAs and their target genes [33].

In China, the main sugarcane variety grown during the past 20 years is ROC22, which is a *Saccharum* spp. hybrid from ROC5 × 69–463 (high-sugar line) and encompasses approximately 60% of the total sugarcane cultivated area. ROC22 is susceptible to *S. scitamineum* and results in a poor ratoon performance. In this study, an intergeneric BC2 hybrid with smut resistant character named YC05–179 (YC01–134 × ROC20) and the smut-susceptible variety ROC22 were used as experimental materials. First, the TaqMan qRT-PCR technology and physiological enzyme activity determination were performed to analyze the proliferation of *S. scitamineum* and activity changes of key enzymes involved in reactive oxygen species metabolic and phenylpropanoid metabolic pathways at various time points in different sugarcane genotypes infected by *S. scitamineum*. And the critical time points during interaction between sugarcane and *S. scitamineum* were determined. Second, the RNA of YC05–179 and ROC22 at different stages of infection was used for degradome sequencing analysis to screen miRNAs and their predicted target genes. Third, the expression level of partial obtained miRNAs and their corresponding predicted target genes was verified by qRT-PCR. The purpose of this study is to understand the miRNA-mediated molecular mechanisms in sugarcane response to *S. scitamineum* stress.

## Methods

### Plant materials and inoculation

The tested sugarcane varieties were YC05–179 (smut resistant) and ROC22 (smut susceptible). The pathogen strain was mixed spores of *S. scitamineum* collected from different sugarcane varieties and different planting areas in the field. They were all provided by the Key Laboratory of Sugarcane Biology and Genetic Breeding, Ministry of Agriculture, Fujian Agriculture and Forestry University (Fuzhou, Fujian, China). The plants were treated with *S. scitamineum* using the method of Su et al. [14]. The consistent and robust cane stems were selected, which were cut into single-bud stem, and then soaked in running water for 2 d. The cane stems were then placed on a tray in a 32 °C incubator (65% relative humidity) with 12 h light/12 h dark conditions for germination. The water was replaced once in the morning and in the evening. When the cane buds had grown to about 2 cm in height, the treatment group were subjected to acupuncture inoculation with 5 × 10^6^ spores/mL *S. scitamineum* spore suspension (0.01% Tween-20) in buds, and the control group underwent acupuncture inoculation with sterile water (0.01% Tween-20). The cane stems were then placed in a 28 °C incubator (65% relative humidity) with 12 h light/12 h dark conditions. The sugarcane bud tissues were collected at 0, 1, 2, 3, 5 and 7 d after inoculation, fixed in liquid nitrogen, and then stored at − 80 °C until analysis.

### Sugarcane genomic DNA and total RNA extraction and quality testing

Three sugarcane buds were taken from each sample. Genomic DNA of the samples was extracted according to the modified CTAB method of Yao et al. [34]. Total RNA was extracted using TRIzol (Invitrogen, Carlsbad, CA, USA). The integrity of total RNA was detected by 1.5% agarose gel electrophoresis. The concentration and purity of genomic DNA and total RNA were assessed using a Nano-Drop (Thermo Fisher, USA) and Agilent 2100 (Agilent Technologies, Palo Alto, CA, USA) systems.

### Quantification of smut pathogen in sugarcane

The quantification of *S. scitamineum* in YC05–179 and ROC22 was detected using TaqMan qRT-PCR technology that was developed by Su et al. [35]. The detection primers were *bE*Q-F: 5’-TGAAAGTTCTCATGCAAGCC-3′ and *bE*Q-R: 5’-TGAGAGGTCGATTGAGGTTG-3′, and the TaqMan probe was 5’-FAM-TGCTCGACGCCAATTCGGAG-TAMRA-3′. A standard curve was established by a 10-fold gradient dilution with recombinant plasmid DNA containing the *bE* gene (b East mating type gene, GenBank Accession No. U61290.1). The concentration of DNA was 500 ng/μL. The amplification program was 50 °C for 2 min; followed by 40 cycles of 95 °C for 10 min, 95 °C for 15 s, 60 °C for 1 min; and a single-point fluorescence detection at 60 °C. The blank control replaces the template DNA with an equal volume of ddH_2_O. The negative control is the DNA of pathogen-free FN41 (a newly released *Saccharum* spp. hybrid from Yuetang91–976 × ROC20 in China) 4-month-old plantlets and the positive control is the genomic DNA of *S. scitamineum*. Each run of TaqMan qRT-PCR contained three replicates. A standard curve was drawn using Microsoft Excel and GraphPad Prism softwares, and the quantification of *S. scitamineum* in each sample was calculated.

### Determination the activity of key enzymes involved in reactive oxygen species metabolic and phenylpropanoid metabolic pathways

At 0, 1, 2, 3, 5 and 7 d after inoculation, 0.1 mol/L borate buffer (pH 8.7) was used for the extraction of crude enzyme solution in cane bud tissues [36]. All samples (five buds per sample) were weighed and homogenized with ice-cold borate buffer at the ratio of 1 g/10 mL, as well as with a small amount of quartz sand and polyvinylpyrrolidone (PVP). The supernatant was centrifuged at 5000 rpm/min for 15 min at 4 °C. The final supernatant was used as the crude enzyme solution for the determination of peroxidase (POD), superoxide dismutase (SOD), catalase (CAT), polyphenol oxidase (PPO), phenylalanine ammonia lyase (PAL), and tyrosine ammonia lyase (TAL) activities. The guaiacol method was used to determine POD enzyme activity [37]. SOD activity was measured using nitroblue tetrazolium (NBT) photoreduction [38]. The activities of CAT, PPO, PAL, and TAL were determined according to the methods of Beers and Sizer [39], Galeazzi et al. [40], Green et al. [41], and Kofalvi and Nassuth [42], respectively. Three biological replicates were prepared for each treatment. The data were analyzed using Microsoft Excel and SPSS softwares. GraphPad Prism software was used to produce figures. Duncan’s method was used for statistical analysis.

### Degradome library construction, sequencing and bioinformatics analysis

The samples for degradome sequencing were YC05–179 and ROC22 cane buds inoculated with sterile water at 0 d (control group) and with *S. scitamineum* at 2 and 5 d (treated group). These samples were named Y0, Y2, Y5, R0, R2, and R5, respectively. The quality of total RNA met the OD_260/280_ of 1.8–2.2, with normal absorption peak at 260 nm, and 28S/18S ratio ≥ 1.5. Library construction, degradome sequencing, and data analysis were commissioned by Beijing BioMed Biotech Co., Ltd. (Beijing, China) using the Illumina HiSeq™ 2500 [43]. Raw reads were generated from degradome sequencing. Then the reads with adapters, the low-quality reads with mass values below 30 and bases in excess of 20%, the reads with unknown base N contents greater than or equal to 10%, and the reads less than 18 nt in length were filtered out to eventually obtain clean reads and cluster reads [31]. Non-coding RNAs (rRNA, scRNA, snoRNA, snRNA, and tRNA) were removed by aligning clean reads and cluster reads with the Rfam database [44]. Because whole genome sequencing of *Saccharum* spp. hybrid has not been completed to date, we mapped the remaining sequences using the bowtie software and targetfinder software to align with the sugarcane reference sequences (sugarcane transcriptome under *S. scitamineum* stress [11], GSS database, and EST database in NCBI) and the known miRNAs from miRBase 21.0 (http://www.mirbase.org) or miRNAs identified in our previous study [14] to predict miRNA target genes. When the score of miRNA that matches the mRNA was less than or equal to 7, the transcript sequence was considered as the miRNA target gene [45, 46].

### miRNA targets prediction and identification

Based on the depth statistics of mRNA target genes and the abundance of transcripts, the target genes were grouped into five categories, namely, 0, 1, 2, 3, and 4 [45, 47]. Category 0 indicated that the position had a depth > 1, an abundance equal to the maximum of the transcript abundance, and the transcript had only one maximum value. Category 1 indicated that the position had a depth > 1, an abundance equal to the maximum value of the transcript abundance, and the transcript had two or more maxima. Category 2 represented the depth of the position was > 1 and the abundance was less than the maximum but higher than the mean of the transcript abundance. Category 3 represented that the depth was > 1 and the abundance was less than or equal to the mean of the transcript abundance. Category 4 represented the depth of the position equal to 1.

The degradation site of predicted target gene was analyzed by Cleaveland software at *p*-value < 0.05. The screened predicted target gene sequences with degradation sites were compared to the COG, GO, KEGG, NR, NT, and Swiss-Port databases to obtain the predicted target gene annotation information. The expression level of the predicted target gene was calculated using the fragments per kilobase of transcript per million mapped reads (FPKM) method which eliminated the influence of gene length and sequencing difference in high-throughput sequencing [48]. The treatment group and the control group were compared to analyze the differential expression of the predicted target genes, and the ratio of expression level was expressed as the fold-change [49]. A fold-change ≥2 and a false discovery rate (FDR) < 0.01 were used as screening criteria for differentially expressed predicted target genes. The FDR was obtained by correcting the difference in the significance of the *p*-value [50]. The *p*-value was corrected using the Benjamini-Hochberg calibration method, and the FDR was used as a screening index to ensure the quality of differentially expressed genes. DY2 and DY5 represent the differentially expressed predicted target genes at 2 and 5 d after YC05–179 was inoculated with *S. scitamineum*, whereas DR2 and DR5 represent the differentially expressed predicted target genes at 2 and 5 d after ROC22 was inoculated with *S. scitamineum*. DY2, DY5, DR2, and DR5 were respectively analyzed by COG, GO, and KEGG to investigate functional and related metabolic pathways of the differentially expressed predicted target genes. The continuously and non-continuously differentially expressed target genes predicted in YC05–179 and ROC22 at different time points were analyzed to reveal potential genes that were related to smut resistance.

### qRT-PCR validation of the expression level of miRNAs and their predicted targets

MiR168a-5p, miR5293, miR160a, nov-miR132, nov-mir-143, nov-mir-63, nov-mir-18, miR5368, nov-mir-10, miR858b, nov-mir-97, miR162a, miR529-3p, and their partial predicted target genes (Table 1) were verified by qRT-PCR. The qRT-PCR primers of the predicted target genes (Additional file 1: Table S1) were designed using Beacon Designer software. Glyceraldehyde-3-phosphate dehydrogenase (*GAPDH*) was used as the internal reference gene [51]. Total RNA of YC05–179 and ROC22 inoculated with sterile water for 0 d and with *S. scitamineum* for 2 and 5 d were digested with RNase-Free DNase (Promega, USA) to remove DNA contamination, followed by reverse transcription to generate first-strand cDNA using a PrimeScript® RT reagent kit (Perfect Real Time) (TaKaRa, Dalian, China). The qRT-PCR reaction system was prepared using the SYBR Green dye method following the instructions of the FastStart Universal SYBR Green PCR Master (ROX) kit (Roche, Shanghai, China). qRT-PCR amplification was performed on an ABI 7500 instrument at 50 °C for 2 min, followed by 40 cycles of 95 °C for 15 s and 60 °C for 1 min. Three replicates were used for each sample. The blank controls were used to replace the cDNA template with sterile water. Stem-loop method was used to detect the expression level of candidate miRNA [52]. According to the method of Varkonyi-Gasic et al. [33], miRNA stem-loop primers (RT primer) and upstream primers (Additional file 1: Table S2) were designed. The downstream primer was a universal primer for anchoring the stem-loop region. *GAPDH* was used as the internal reference gene [53]. Total RNA was reverse-transcribed using an Applied Biosystems® TaqMan® MicroRNA Reverse Transcription kit (Invitrogen, Carlsbad, CA, USA) with a 15-μL reverse-transcription system. In the process of reverse transcription, when using cDNA template as internal control, the RT primers were replaced by random primers. The miRNA qRT-PCR reaction system and procedure were the same as that for the predicted target gene. qRT-PCR data were calculated using the 2^−ΔΔCT^ method [54].

**Table 1:** The basic information of selected miRNAs and their corresponding predicted target genes for qRT-PCR validation

No.	miRNA name	Predicted target gene ID	Predicted target gene annotation	Cleavage site	Category
1	miR168a-5p	Sugarcane_Unigene_BMK.66779	Protein argonaute 1B (*AGO 1B*)	984	0
2	miR5293	Sugarcane_Unigene_BMK.40335	Auxin-induced protein (*AIP*)	720	1
3	miR160a	Sugarcane_Unigene_BMK.63027	Auxin response factor 8 (*ARF8*)	879	0
4	nov-mir-132	Sugarcane_Unigene_BMK.28594	Cinnamoyl-CoA reductase (*CCR*)	588	1
5	nov-mir-143	Sugarcane_Unigene_BMK.64656	Ethylene insensitive 3-like 3 protein (*EIL3*)	1740	1
6	nov-mir-63	Sugarcane_Unigene_BMK.75694	Glycerol kinase (*GK*)	1790	0
7	miR396e-5p	Sugarcane_Unigene_BMK.60551	Growth-regulating factor 8 (*GRF8*)	465	0
8	nov-mir-66	Sugarcane_Unigene_BMK.60551	Growth-regulating factor 8 (*GRF8*)	465	0
9	nov-mir-18	Sugarcane_Unigene_BMK.60551	Growth-regulating factor 8 (*GRF8*)	465	0
10	miR5368	Sugarcane_Unigene_BMK.51989	Hypersensitive-induced response protein 1 (*HIR1*)	866	1
11	nov-mir-10	Sugarcane_Unigene_BMK.62668	Mildew resistance locus o (*MLO*)	510	1
12	miR858b	gi35098237	Myb-related protein Hv33 (*MYB2*)	524	1
13	nov-mir-97	Sugarcane_Unigene_BMK.51113	Protein phosphatase 2C (*PP2C*)	192	1
14	miR162a	Sugarcane_Unigene_BMK.67816	S-adenosylmethionine decarboxylase (*SAMDC*)	1696	2
15	miR529-3p	Sugarcane_Unigene_BMK.64654	Ubiquitin carboxyl-terminal hydrolase isozyme L5-like (*UCH-L5*)	864	0

Cleavage site, nucleotide number from 5′ end of cDNA; Category, the “category” of this cleaveage site

## Results

### Smut pathogen proliferation and changes in the activity of key enzymes involved in reactive oxygen species metabolic and phenylpropanoid metabolic pathways during the early stage of infection

**Fig. 1:**
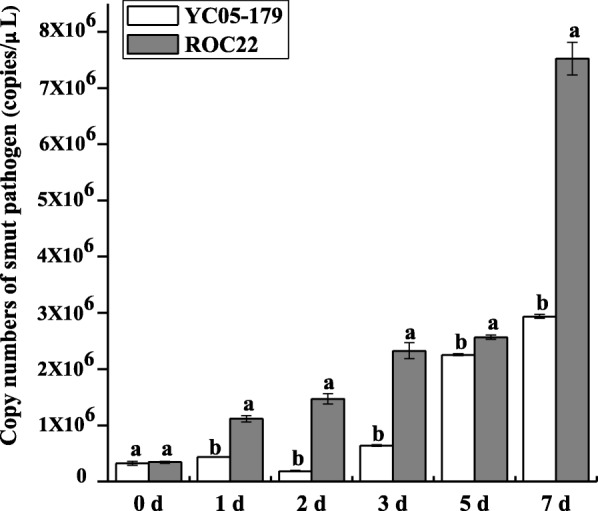
The amount of smut pathogen in YC05–179 and ROC22 inoculated with *Sporisorium scitamineum* by TaqMan qRT-PCR analysis. The quantification of smut pathogen were calculated with the equation of the linear regression line. All data points were means ± standard error (*n* = 3). Different lowercase letters indicated a significant difference between resistant and susceptible genotypes, as determined by the Duncan’s new multiple range test (*p* < 0.05). YC05–179, smut-resistant genotype; ROC22, smut-susceptible genotype

The results of TaqMan qRT-PCR (Fig. 1) showed that the Ct values of smut-resistant genotype (YC05–179) and -susceptible genotype (ROC22) inoculated with sterile water, negative control, and blank control were all higher than 37, indicating the absence of *S. scitamineum*, whereas the Ct value of samples inoculated with *S. scitamineum* was between 27 and 33. The quantification of *S. scitamineum* in YC05–179 and ROC22 increased with inoculation time, which were 323,995.15 ± 53,563.55 copies/μL–2,935,184.09 ± 36,789.33 copies/μL and 340,733.51 ± 29,137.42 copies/μL–7,525,544.93 ± 358,488.58 copies/μL, respectively. At 0 d after inoculation, the quantification of *S. scitamineum* was similar in YC05–179 and ROC22. The dynamic increase in DNA copy number of the *S. scitamineum* in ROC22 was more distinct at 1, 2 and 3 d after inoculation, which was 3.27-, 4.31-, and 6.82-fold respectively of that at 0 d after inoculation. The quantification of *S. scitamineum* in YC05–179 was increased at 1 d but decreased at 2 d, which was 1.34-fold and 0.57-fold that at 0 d, respectively. At 5 and 7 d, the content of *S. scitamineum* in YC05–179 showed minimal change, which was 6.96- and 9.06-fold that at 0 d, whereas the content of *S. scitamineum* in ROC22 continued to increase and peaked at 7 d, which was 22.09-fold that at 0 d after inoculation.

**Fig. 2:**
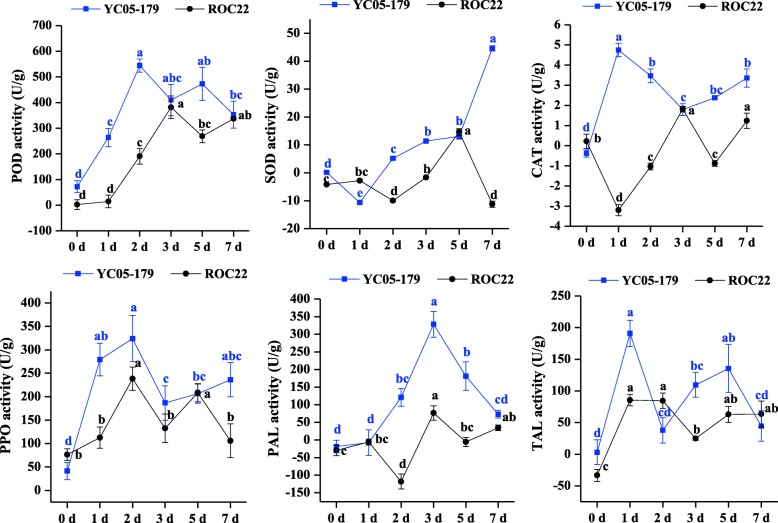
Variations of the activity of key enzymes involved in reactive oxygen species metabolic and phenylpropanoid metabolic pathways during the early stage of *Sporisorium scitamineum* infection. All data points were means ± standard error (*n* = 3). Different lowercase letters indicated a significant difference, as determined by the Duncan’s new multiple range test (*p* < 0.05). YC05–179, smut-resistant genotype; ROC22, smut-susceptible genotype; POD, peroxidase; SOD, superoxide dismutase; CAT, catalase; PPO, polyphenol oxidase; PAL, phenylalnine ammonialyase; TAL, tyrosine ammonia-lyase

Figure 2 showed the activity changes of six key enzymes involved in reactive oxygen species metabolic and phenylpropanoid metabolic pathways in two sugarcane genotypes post inoculation with *S. scitamineum*. After inoculation, the activity of POD in YC05–179 and ROC22 was all increased. It peaked at 2, 3 and 5 d in YC05–179, whereas at 3 and 7 d in ROC22. The activity of SOD in YC05–179 was decreased at 1 d but gradually increased and reached a peak value at 7 d. In ROC22, the activity of SOD was stable at 1 d, and decreased at 2 and 7 d, whereas reached a peak value at 5 d. There was an opposite expression pattern in the activity of CAT between YC05–179 and ROC22. After inoculation with *S. scitamineum*, the activity of CAT in YC05–179 was significantly increased and peaked at 1 d, follow by 2, 5 and 7 d, whereas that in ROC22 was decreased at 1, 2 and 5 d. PAL and TAL, which are upstream of the phenylpropanoid metabolic pathway, inhibit lignin biosynthesis when their activities are inhibited [55]. Inoculation of YC05–179 with *S. scitamineum* resulted in a stronger activity of PAL at 3 d, followed by 2 and 5 d. The activity of PAL in ROC22 was increased at 3 and 7 d but decreased at 2 and 5 d. In YC05–179, the activity of TAL reached a peak value at 1 and 5 d, followed by 3 d, and remained stable at 2 and 7 d. The activity of TAL in ROC22 were all increased after inoculation and peaked at 1, 2, 5 and 7 d. PPO, which catalyzes the phenols to quinones, was assumed to be involved in plant defense against pathogens [56]. After inoculation, the activity of PPO in YC05–179 was significantly increased and peaked at 1, 2 and 7 d, followed by 3 and 5 d. The activity of PPO in ROC22 was significantly increased at 2 and 5 d but remained stable at the other time points. In summary, the activities of POD, SOD, CAT, PPO, PAL and TAL in YC05–179 were generally higher than those in the control (0 d) and ROC22 plants after inoculation with *S. scitamineum*. At 2 and 5 d, the metabolic levels of activated oxygen and phenylpropanoid in YC05–179 were stronger than those in ROC22. Furthermore, there was a significant difference in smut pathogen content between YC05–179 and ROC22 after inoculation (Fig. 1). At the early stage of 2 d, the amount of *S. scitamineum* was decreased in YC05–179 but increased in ROC22 (Fig. 1). Therefore, we used 2 and 5 d after inoculation with *S. scitamineum* as the best sampling time points for degradome sequencing.

### Degradome library construction and data summary

Six cane-bud samples, including YC05–179 and ROC22 inoculated with sterilized water for 0 d and those inoculated with *S. scitamineum* for 2 and 5 d, were sequenced. Each sample was generated with no less than 10 M reads, and a total of 122.33 M raw reads in six samples was gained. After data evaluation, clean reads and cluster reads were obtained with a length of 47 nt. The number of clean reads in the Y0, Y2, Y5, R0, R2, and R5 libraries was 18,662,178, 21,772,014, 22,928,020, 19,440,129, 18,537,103, and 20,995,053, respectively (Table 2). After comparing the clean reads and cluster tags with Rfam database to exclude rRNAs, tRNAs, snoRNAs, and snRNA (except for miRNAs), the remaining sequences were aligned with the reference sequence of sugarcane to obtain the fully mapped data, which consisted of 2,748,695 (Y0), 3,333,316 (Y2), 3,446,802 (Y5), 2,490,391 (R0), 3,151,567 (R2), and 3,001,075 (R5) reads (Table 2) that were then used in the analysis of degradation sites.

**Table 2:** Statistic results of degradome sequencing data

Sample name	Total reads	Tags number	Tags percent	Clean reads	Q30	Clean reads after Rfam alignment	Mapped data	Mapped data percentage
Y0	18,665,458	3280	0.02%	18,662,178	94.29%	6,613,553	2,748,695	41.56%
Y2	21,777,063	5049	0.02%	21,772,014	94.45%	8,270,381	3,333,316	40.30%
Y5	22,944,082	16,062	0.07%	22,928,020	94.59%	8,609,667	3,446,802	40.03%
R0	19,451,881	11,752	0.06%	19,440,129	93.73%	6,342,529	2,490,391	39.26%
R2	18,549,222	12,119	0.07%	18,537,103	94.56%	7,226,017	3,151,567	43.61%
R5	20,999,522	4469	0.02%	20,995,053	94.46%	7,007,751	3,001,075	42.83%

Total reads, total raw reads of sequencing; Tags number, reads number with adaptor; Tags percent, proportion of reads with adaptors in total reads; Clean reads, clean reads numbers after filtering; Q30, percentage of Q30; Clean reads after Rfam alignment, reads numbers of samples compared with sugarcane reference sequences (sugarcane transcriptome under smut pathogen stress [11], GSS database, and EST database in NCBI); Mapped data, reads numbers of samples compared to sugarcane reference sequences; Mapped data percentage, percentage of mapped numbers in the clean reads after Rfam alignment. Y0 and R0 mean the genes in the libraries of YC05–179 and ROC22 inoculated with sterile water at 0 d, respectively. Y2 and Y5 mean the genes in the libraries of YC05–179 inoculated with *Sporisorium scitamineum* at 2 and 5 d, respectively. R2 and R5 mean the genes in the libraries of ROC22 inoculated with *S. scitamineum* at 2 and 5 d, respectively. YC05–179, smut-resistant genotype; ROC22, smut-susceptible genotype

### Degradation site specificity and diversity

**Fig. 3:**
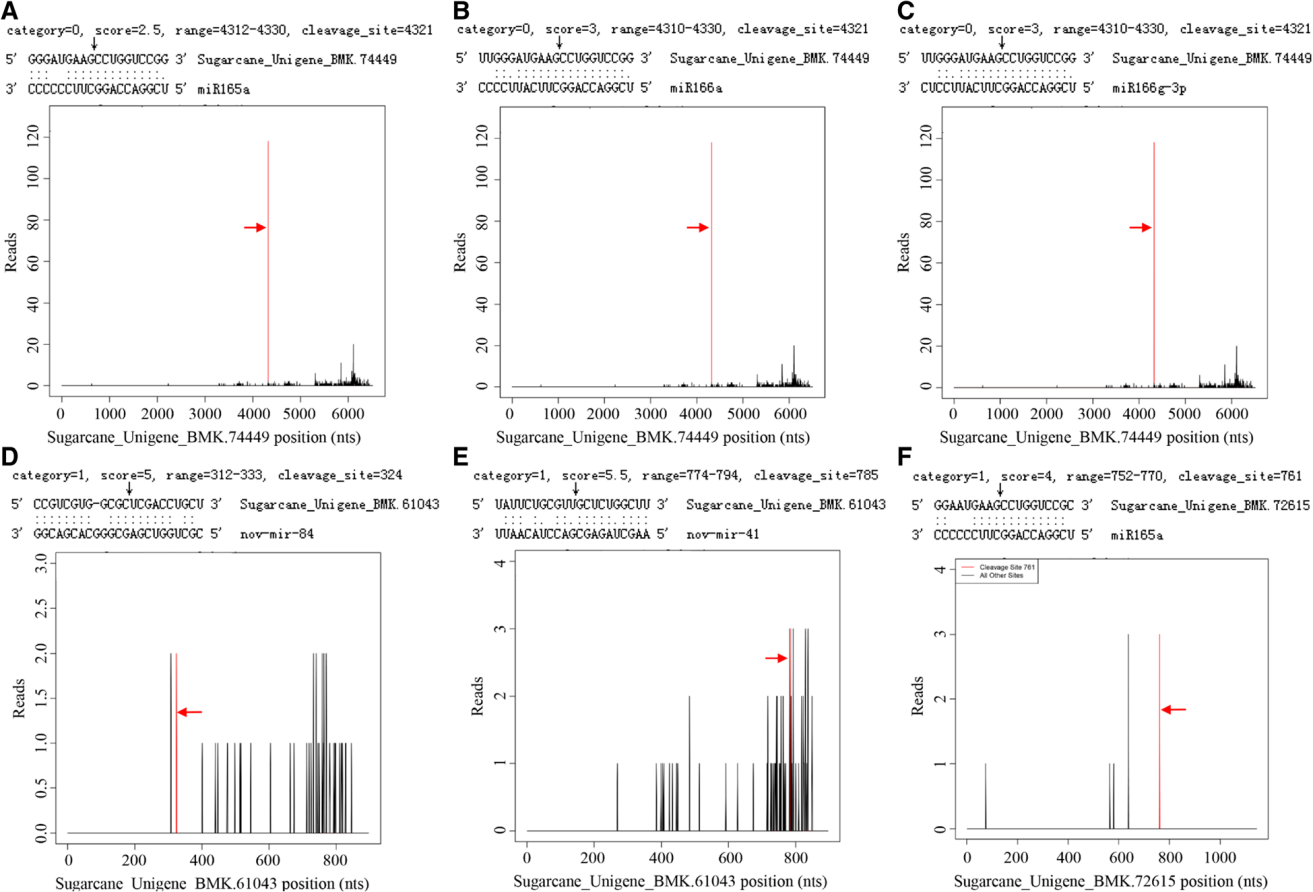
T-plots of the predicted targets cleaved by miRNAs confirmed by degradome sequencing. The alignment along with the detected cleavage frequencies (absolute numbers) were shown beside the black arrow and it showed the miRNA with a portion of its target sequence (top). The two dots indicated matched RNA base pairs, and one dot showed a GU mismatch whereas none dot represent other types of mismatch. Category 0 indicated that the position had a depth > 1, an abundance equal to the maximum of the transcript abundance, and the transcript had only one maximum value; Category 1 indicated that the position had a depth > 1 and an abundance equal to the maximum value of the transcript abundance, and the transcript had two or more maxima [45, 47]. The T-plots showed the distribution of 3′ end of the degradome tags within the full-length of the predicted target mRNA sequence (bottom). The red line represented the cleaved target tags and was shown in red arrow. **a**–**c** T-plots of Sugarcane_Unigene_BMK.74449 cleaved simultaneously by miR165a, miR166a, and miR166g-3p at position 4321. **d** and **e** T-plots of Sugarcane_Unigene_BMK.61043 cleaved by nov-mir-84 and nov-mir-41 at positions 324 and 785, respectively. **f** T-plot of Sugarcane_Unigene_BMK.72615 cleaved by miR165a at position 761

According to sequence homology, each miRNA can simultaneously target two or more target genes belonging to the same type or having similar conserved domains, and a target gene can also be cleaved by multiple miRNAs [57, 58]. In this study, the degradome sequencing results showed that the predicted target gene could be cleaved by different miRNAs at a specific cleavage site. For example, Sugarcane_Unigene_BMK.74449 could be cleaved simultaneously by miR165a, miR166a, and miR166g-3p at position 4321 (Figs. 3a, b, c). The predicted target gene could also be cleaved by different miRNAs at different cleavage sites, e.g., Sugarcane_Unigene_BMK.61043 could be cleaved by nov-mir-84 and nov-mir-41 at positions 324 and 785, respectively (Figs. 3d, e). In addition, multiple target genes could be cleaved by the same miRNA. For example, Sugarcane_Unigene_BMK.74449 and Sugarcane_Unigene_BMK.72615 could be cleaved by miR165a (Figs. 3a, f).

### Target gene identification, annotation, and classification

**Fig. 4:**
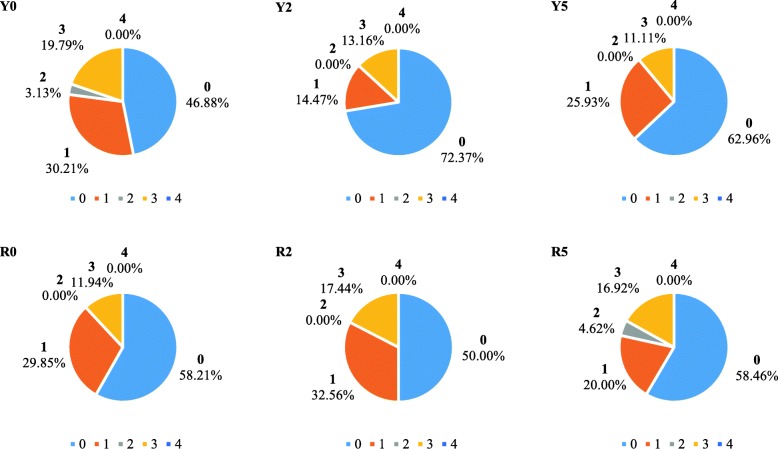
The classification of the predicted target genes in the six libraries. Y0 and R0 mean the predicted target genes in the libraries of YC05–179 and ROC22 inoculated with sterile water at 0 d, respectively. Y2 and Y5 mean the predicted target genes in the libraries of YC05–179 inoculated with *Sporisorium scitamineum* for 2 d and 5 d, respectively. R2 and R5 mean the predicted target genes in the libraries of ROC22 inoculated with *S. scitamineum* for 2 d and 5 d, respectively

Based on the miRNA database [14] and Unigene database [11] of sugarcane after smut pathogen infection, 2922 miRNA-mRNA pairs were screened from six libraries using targetfinder software. A total of 337 degradation sites were detected by Cleaveland software, corresponding to 219 miRNAs (97 known miRNAs and 112 new miRNAs) and 309 predicted target mRNAs (Additional file 2: Table S3). The predicted target mRNAs were all mainly classified as Category 0 in all six libraries, without Category 4 (Fig. 4). We found that the predicted target genes cleaved by miRNAs, such as squamosa promoter-binding-like protein (*SPL*), no apical meristem (*NAM*), v-myb avian myeloblastosis viral oncogene homolog (*MYB*), auxin response factor, extensin-like protein, somatic embryogenesis receptor kinase (*SERK*), *CAT*, ethylene-insensitive 3-like 3 protein (*EIL3*), and miRNA precursor encoding genes, may be involved in various life-controlling processes of sugarcane.

### COG, GO and KEGG analyses of differentially expressed predicted target genes

Differentially expressed predicted target genes were screened according to the criteria of fold-change ≥2 and FDR < 0.01. The results showed that 69 predicted target genes (31 upregulated and 38 downregulated) of DY2 and 73 predicted target genes (37 upregulated and 36 downregulated) of DY5 were differentially expressed in YC05–179. A total of 144 predicted target genes (70 upregulated and 74 downregulated) of DR2 and 138 predicted target genes (71 upregulated and 67 downregulated) of DR5 were differentially expressed in ROC22. In conclusion, the total number of upregulated predicted target genes was similar to that of downregulated predicted target genes at both 2 and 5 d in either YC05–179 or ROC22.

COG analysis showed that the differentially expressed predicted target genes in DY2, DY5, DR2 and DR5 were functionally annotated to the categories of signal transduction mechanisms (1, 0, 2, 0), energy production and conversion (2, 1, 2, 4), and inorganic ion transport and metabolism (1, 1, 2, 3), etc. (Additional file 1: Figure S1). GO enrichment results showed that the differentially expressed predicted target genes of DY2, DY5, DR2, and DR5 were involved in 28, 31, 31 and 33 GO categories, respectively (Table 3), of which highly enriched GO categories in terms of cellular function were cell (35, 33, 85, 85), membrane (18, 17, 29, 36), organelle (30, 31, 79, 80), and cell part (35, 33, 85, 85). GO categories that were highly enriched in terms of molecular function were catalytic activity (20, 12, 39, 40), and binding (25, 26, 57, 53). GO categories with more enriched biological process were metabolic process (21, 19, 50, 56), cellular process (22, 23, 60, 62), single-organism process (21, 18, 47, 49), response to stimulus (12, 8, 27, 30), and biological regulation (16, 12, 34, 35).

**Table 3:** Gene ontology based on differentially expressed predicted target genes

Category	GO term	Differentially expressed predicted target genes				Total genes
		DY2	DY5	DR2	DR5	
Cellular component	extracellular region	1	1	6	3	974
	cell	35	33	85	85	24,549
	membrane	18	17	29	36	9992
	cell junction	0	1	2	2	385
	macromolecular complex	4	5	14	16	3200
	organelle	30	31	79	80	21,904
	organelle part	4	8	20	22	4646
	membrane part	9	5	9	11	4082
	cell part	35	33	85	85	24,618
Molecular function	nucleic acid binding transcription factor activity	3	5	11	10	1052
	catalytic activity	20	12	39	40	17,825
	receptor activity	0	0	1	1	222
	structural molecule activity	0	1	3	6	753
	transporter activity	3	2	3	2	1876
	binding	25	26	57	53	20,072
	electron carrier activity	0	0	0	4	834
	antioxidant activity	0	1	0	1	370
	enzyme regulator activity	1	0	1	0	344
	molecular transducer activity	0	0	1	1	456
Biological process	reproduction	1	1	0	1	503
	immune system process	2	3	2	4	454
	metabolic process	21	19	50	56	21,790
	cellular process	22	23	60	62	19,163
	reproductive process	3	3	7	9	1722
	signaling	2	1	8	8	1423
	multicellular organismal process	6	4	14	15	2556
	developmental process	8	5	16	19	3341
	growth	2	1	5	4	595
	single-organism process	21	18	47	49	14,355
	rhythmic process	0	1	0	0	59
	response to stimulus	12	8	27	30	7834
	localization	7	9	19	14	4192
	multi-organism process	1	2	4	6	1376
	biological regulation	16	12	34	35	6552
	cellular component organization or biogenesis	6	3	14	12	3785

DY2 and DY5 represent the differentially expressed predicted target genes at 2 and 5 d after YC05–179 was inoculated with *Sporisorium scitamineum*, whereas DR2 and DR5 represent the differentially expressed predicted target genes at 2 and 5 d after ROC22 was inoculated *S. scitamineum*. YC05–179, smut-resistant genotype; ROC22, smut-susceptible genotype

Table 4 showed the results of KEGG pathway enrichment in YC05–179 and ROC22 post inoculation with *S. scitamineum*. The differentially expressed predicted target genes involved in disease resistance-related metabolic pathways in YC05–179 and ROC22 mainly belonged to the categories of plant hormone signal transduction, ubiquinone and other terpenoid-quinone biosynthesis, ubiquitin mediated proteolysis, plant-pathogen interaction, oxidative phosphorylation, peroxisome, phenylalanine, tyrosine and tryptophan biosynthesis, and phagosome. Among them, the differentially expressed predicted target genes involved in plant-pathogen interaction were only found in YC05–179, whereas the differentially expressed predicted target genes involved in phenylalanine, tyrosine, and tryptophan biosynthesis and phagosome were only found in ROC22.

**Table 4:** KEGG enrichment results of differentially expressed predicted target genes in YC05–179 and ROC22 inoculated with *Sporisorium scitamineum* for 2 and 5 d

Library	Kegg_pathway	ko_id	Cluter_frequency	*P*-value	Corrected_*P*-value	Enrichment_factor
DY2	Plant hormone signal transduction	ko04075	2 out of 9, 22.22%	0.071009	0.710085	0.22
	Ubiquinone and other terpenoid-quinone biosynthesis	ko00130	1 out of 9, 11.11%	0.071806	0.71806	0.07
	Glycerolipid metabolism	ko00561	1 out of 9, 11.11%	0.126198	1	0.13
	Photosynthesis	ko00195	1 out of 9, 11.11%	0.137578	1	0.15
	Arginine and proline metabolism	ko00330	1 out of 9, 11.11%	0.199175	1	0.22
	Ubiquitin mediated proteolysis	ko04120	1 out of 9, 11.11%	0.213623	1	0.24
	Plant-pathogen interaction	ko04626	1 out of 9, 11.11%	0.235498	1	0.26
	Cysteine and methionine metabolism	ko00270	1 out of 9, 11.11%	0.236767	1	0.27
	Oxidative phosphorylation	ko00190	1 out of 9, 11.11%	0.313078	1	0.37
	RNA transport	ko03013	1 out of 9, 11.11%	0.371941	1	0.45
	SNARE interactions in vesicular transport	ko04130	1 out of 10, 10%	0.055905	0.670858	0.06
DY5	Plant hormone signal transduction	ko04075	2 out of 10, 20%	0.085911	1	0.25
	Tryptophan metabolism	ko00380	1 out of 10, 10%	0.089401	1	0.09
	Pentose and glucuronate interconversions	ko00040	1 out of 10, 10%	0.092691	1	0.10
	Glycerolipid metabolism	ko00561	1 out of 10, 10%	0.139209	1	0.15
	Photosynthesis	ko00195	1 out of 10, 10%	0.151658	1	0.16
	Fructose and mannose metabolism	ko00051	1 out of 10, 10%	0.154745	1	0.17
	Peroxisome	ko04146	1 out of 10, 10%	0.201316	1	0.22
	Ubiquitin mediated proteolysis	ko04120	1 out of 10, 10%	0.234362	1	0.26
	Plant-pathogen interaction	ko04626	1 out of 10, 10%	0.257991	1	0.29
	RNA transport	ko03013	1 out of 10, 10%	0.403603	1	0.50
	Ribosome	ko03010	1 out of 10, 10%	0.544618	1	0.76
DR2	Plant hormone signal transduction	ko04075	4 out of 24, 16.67%	0.029416	0.705974	0.30
	Phenylalanine, tyrosine and tryptophan biosynthesis	ko00400	2 out of 24, 8.33%	0.029491	0.707793	0.14
	Photosynthesis	ko00195	2 out of 24, 8.33%	0.05751	1	0.20
	Histidine metabolism	ko00340	1 out of 24, 4.17%	0.117722	1	0.12
	SNARE interactions in vesicular transport	ko04130	1 out of 24, 4.17%	0.129112	1	0.14
	Ubiquitin mediated proteolysis	ko04120	2 out of 24, 8.33%	0.13054	1	0.32
	Cysteine and methionine metabolism	ko00270	2 out of 24, 8.33%	0.157229	1	0.35
	Fatty acid biosynthesis	ko00061	1 out of 24, 4.17%	0.204988	1	0.23
	Pentose and glucuronate interconversions	ko00040	1 out of 24, 4.17%	0.208438	1	0.23
	Porphyrin and chlorophyll metabolism	ko00860	1 out of 24, 4.17%	0.222097	1	0.25
	Ascorbate and aldarate metabolism	ko00053	1 out of 24, 4.17%	0.232192	1	0.26
	Oxidative phosphorylation	ko00190	2 out of 24, 8.33%	0.256669	1	0.49
	Glycine, serine and threonine metabolism	ko00260	1 out of 24, 4.17%	0.283948	1	0.33
	Pentose phosphate pathway	ko00030	1 out of 24, 4.17%	0.290177	1	0.34
	Fructose and mannose metabolism	ko00051	1 out of 24, 4.17%	0.332351	1	0.40
	Arginine and proline metabolism	ko00330	1 out of 24, 4.17%	0.447393	1	0.58
	Phagosome	ko04145	1 out of 24, 4.17%	0.454667	1	0.60
	Ribosome biogenesis in eukaryotes	ko03008	1 out of 24, 4.17%	0.478256	1	0.64
	Amino sugar and nucleotide sugar metabolism	ko00520	1 out of 24, 4.17%	0.478256	1	0.64
	Carbon fixation in photosynthetic organisms	ko00710	1 out of 24, 4.17%	0.507463	1	0.70
	Starch and sucrose metabolism	ko00500	1 out of 24, 4.17%	0.513977	1	0.71
	Ribosome	ko03010	2 out of 24, 8.33%	0.551296	1	0.91
	Glycolysis / Gluconeogenesis	ko00010	1 out of 24, 4.17%	0.636432	1	0.99
	Protein processing in endoplasmic reticulum	ko04141	1 out of 24, 4.17%	0.67661	1	1.10
DR5	Photosynthesis	ko00195	3 out of 24, 12.5%	0.006618	0.132362	0.13
	Plant hormone signal transduction	ko04075	4 out of 24, 16.67%	0.029416	0.588312	0.30
	Ribosome	ko03010	4 out of 24, 16.67%	0.10279	1	0.45
	Selenocompound metabolism	ko00450	1 out of 24, 4.17%	0.106189	1	0.11
	Histidine metabolism	ko00340	1 out of 24, 4.17%	0.117722	1	0.12
	Ubiquitin mediated proteolysis	ko04120	2 out of 24, 8.33%	0.13054	1	0.32
	Cysteine and methionine metabolism	ko00270	2 out of 24, 8.33%	0.157229	1	0.35
	6--Sulfur metabolism	ko00920	1 out of 24, 4.17%	0.169683	1	0.18
	Ubiquinone and other terpenoid-quinone biosynthesis	ko00130	1 out of 24, 4.17%	0.180429	1	0.20
	Tryptophan metabolism	ko00380	1 out of 24, 4.17%	0.201523	1	0.22
	Pentose and glucuronate interconversions	ko00040	1 out of 24, 4.17%	0.208438	1	0.23
	Phenylalanine, tyrosine and tryptophan biosynthesis	ko00400	1 out of 24, 4.17%	0.238852	1	0.27
	Oxidative phosphorylation	ko00190	2 out of 24, 8.33%	0.256669	1	0.49
	Glycine, serine and threonine metabolism	ko00260	1 out of 24, 4.17%	0.283948	1	0.33
	Fructose and mannose metabolism	ko00051	1 out of 24, 4.17%	0.332351	1	0.40
	Peroxisome	ko04146	1 out of 24, 4.17%	0.417358	1	0.53
	Arginine and proline metabolism	ko00330	1 out of 24, 4.17%	0.447393	1	0.58
	Phagosome	ko04145	1 out of 24, 4.17%	0.454667	1	0.60
	Purine metabolism	ko00230	1 out of 24, 4.17%	0.644512	1	1.01
	Protein processing in endoplasmic reticulum	ko04141	1 out of 24, 4.17%	0.67661	1	1.10

DY2 and DY5 represent the differentially expressed predicted target genes at 2 d and 5 d after YC05–179 was inoculated with *S. scitamineum*, whereas DR2 and DR5 represent the differentially expressed predicted target genes at 2 d and 5 d after ROC22 was inoculated *S. scitamineum*. YC05–179, smut-resistant genotype; ROC22, smut-susceptible genotype

### Identification of potential resistance-related target gene

#### Continuous differential expression of predicted target genes common to YC05–179 and ROC22

**Fig. 5:**
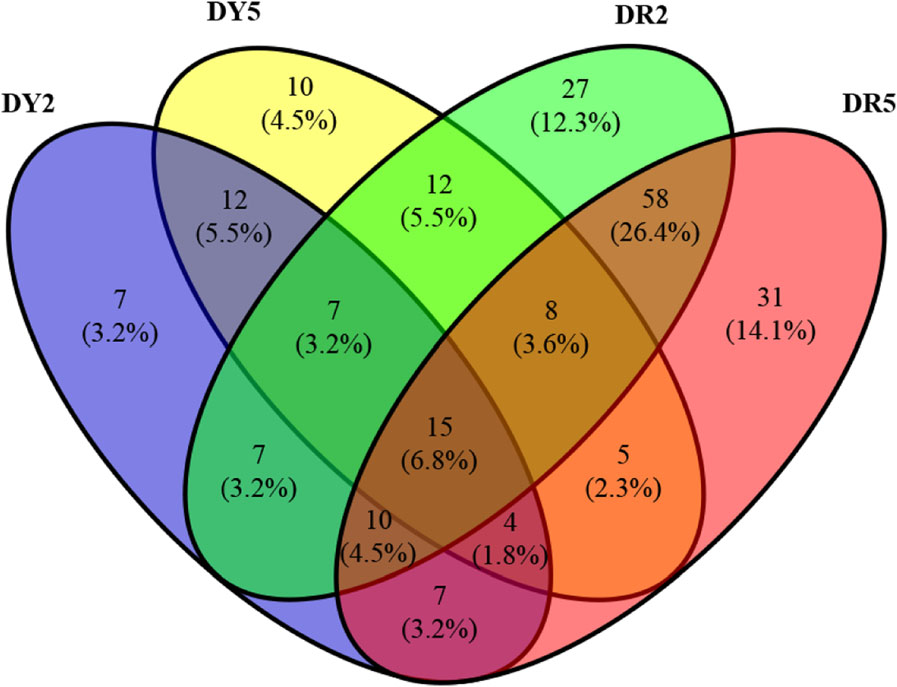
Venn diagram of differentially expressed predicted target genes in YC05–179 (DY) and ROC22 (DR) inoculated with *Sporisorium scitamineum* for 2 d and 5 d. The number and percentage was the quantity and proportion of differentially expressed predicted target genes in library specific or common, respectively. YC05–179, smut-resistant genotype; ROC22, smut-susceptible genotype

Venn diagram analysis of DY2, DY5, DR2, and DR5 showed that there were 38 continuously differentially expressed predicted target genes in YC05–179 at 2–5 d after infection with *S. scitamineum*, of which 91 genes were in ROC22 (Fig. 5). Comparative analysis showed that 15 continuously differentially expressed predicted target genes were shared by YC05–179 and ROC22, such as Sugarcane_Unigene_BMK.64130 (transcription factor GAMYB), gi35264535 (E3 ubiquitin-protein ligase), Sugarcane_Unigene_BMK.51816 (Formin-like protein 5), Sugarcane_Unigene_BMK.52182 (protein argonaute 1D), Sugarcane_Unigene_BMK.51113 (protein phosphatase 2C, PP2C), and Sugarcane_Unigene_BMK.40335 (auxin-induced protein), etc.. If these genes are involved in the pathogenicity-related pathways of plants, then they may be considered as candidate genes for smut-resistance research in the further.

#### Continuous differential expression of predicted target genes specific to YC05–179 or ROC22

Figure 5 showed that there were 12 predicted target genes that were differentially expressed only after YC05–179 was inoculated with *S. scitamineum*, namely, Sugarcane_Unigene_BMK.45107 (Myb-related protein), gi36066484 (Aquaporin SIP1–2), Sugarcane_Unigene_BMK.75694 (glycerol kinase), Sugarcane_Unigene_BMK.52252 (cell wall-associated hydrolase), gi36009271 (nuclear pore complex protein NUP133), Sugarcane_Unigene_BMK.75849 (protein Rf1, mitochondrial precursor), Sugarcane_Unigene_BMK.59405 (nuclear transport factor 2), Sugarcane_Unigene_BMK.41321 (hypothetical protein), gi34973960 (pleckstrin homology domain-containing protein 1), Sugarcane_Unigene_BMK.39426 (hypothetical protein), Sugarcane_Unigene_BMK.2023 (uncharacterized protein LOC101772317 isoform X1) and Sugarcane_Unigene_BMK.33875 (uncharacterized protein LOC113064467). After inoculation, 58 predicted target genes were differentially expressed only in ROC22, which were gi35049661 (SERK2), gi35329294 (auxin response factor 14, ARF14), Sugarcane_Unigene_BMK.71301 (ARF17), gi35090530 (ubiquitin-conjugating enzyme E2 5, UBC5), Sugarcane_Unigene_BMK.54856 (S-adenosylmethionine synthase 1), Sugarcane_Unigene_BMK.64656 (EIL3), and Sugarcane_Unigene_BMK.53115 (oxygen-evolving enhancer protein 2), etc.. These genes could be used as candidates for the further smut-resistance research.

#### YC05–179 continuously, ROC22 non-continuously differentially expressed predicted target genes

A comparative analysis of continuously differentially expressed predicted target genes in YC05–179 and non-continuously differentially expressed predicted target genes in ROC22 was performed to obtain predicted target genes with transient response or lagged response to *S. scitamineum* in ROC22 (Fig. 5). Among these, seven predicted target genes Sugarcane_Unigene_BMK.3158 (formin-like protein 16), Sugarcane_Unigene_BMK.49383 (tRNA-splicing endonuclease subunit Sen2), Sugarcane_Unigene_BMK.56064 (heterogeneous nuclear ribonucleoprotein R-like), Sugarcane_Unigene_BMK.57076 (hypothetical protein), Sugarcane_Unigene_BMK.51607 (Zinc finger MYM-type protein), gi34944423 (Sn1-specific diacylglycerol lipase beta), and Sugarcane_Unigene_BMK.45288 (serine/arginine repetitive matrix protein 2) were shared between continuously differentially expressed predicted target genes in YC05–179 and non-continuously differentially expressed predicted target genes in DR2. Sugarcane_Unigene_BMK.51989 (hypersensitive-induced response protein 1, HIR1), gi36030075 (lysine histidine transporter 1, LHT1), Sugarcane_Unigene_BMK.70126 (E3 ubiquitin-protein ligase SDIR1), and Sugarcane_Unigene_BMK.62668 (MLO-like protein 13) were shared between continuously differentially expressed predicted target genes in YC05–179 and non-continuously differentially expressed predicted target genes in DR5.

#### Non-continuously differentially expressed predicted target genes specific to YC05–179

Figure 5 showed that in YC05–179, in addition to 12 species-specific continuously differentially expressed predicted target genes, there were seven predicted target genes that were differentially expressed only at 2 d after inoculation, including Sugarcane_Unigene_BMK.64437 (NAC domain-containing protein 21/22), and gi35098237 (Myb-related protein), etc.. In addition, 10 predicted target genes were differentially expressed only at 5 d after inoculation, including Sugarcane_Unigene_BMK.71328 (40S ribosomal protein), Sugarcane_Unigene_BMK.75459 (proline-rich receptor-like protein kinase, PERK2), and Sugarcane_Unigene_BMK.44399 (phosphate carrier protein), etc.. As for those genes differentially expressed only in the resistant variety, they could be considered as candidate smut-resistance genes that require further studies.

### qRT-PCR validation of the expression level of miRNAs and their corresponding predicted target genes

**Fig. 6:**
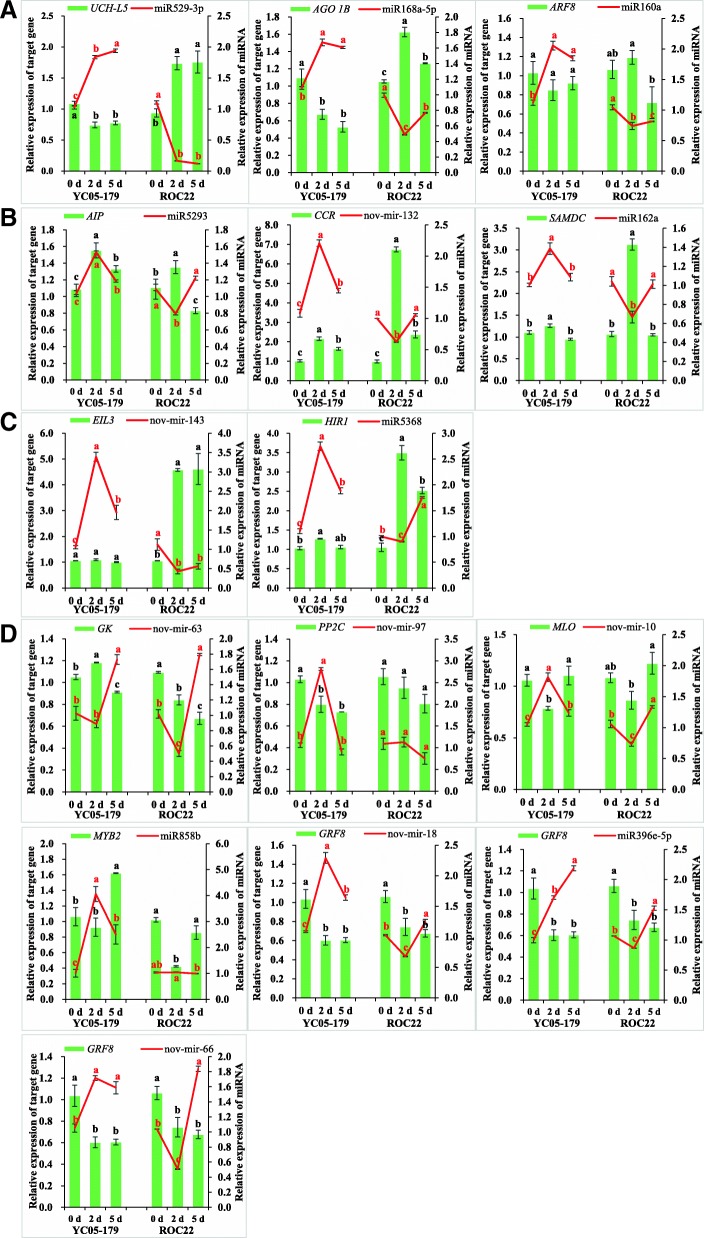
qRT-PCR validation of the expression level of predicted target genes and corresponding miRNAs under *Sporisorium scitamineum* stress at different process times. **a** Expression analysis of miR529-3p, miR168a-5p, miR160a, and their predicted target genes *UCH-L5*, *AGO 1B*, and *ARF8*. **b** Expression analysis of miR5293, nov-mir-132, miR162a, and their predicted target genes *AIP*, *CCR*, and *SAMDC*. **c** Expression analysis of nov-mir-143, miR5368, and their predicted target genes *EIL3* and *HIR1*. **d** Expression analysis of nov-mir-63, nov-mir-97, nov-mir-10, miR858b, nov-mir-18, miR396e-5p, nov-mir-66, and their predicted target genes *GK*, *PP2C*, *MLO*, *MYB2*, and *GRF8*. All data points were means ± standard error (*n* = 3). Different lowercase letters indicated a significant difference, as determined by the Duncan’s new multiple range test (*p* < 0.05). YC05–179, smut-resistant genotype; ROC22, smut-susceptible genotype. *UCH-L5*, ubiquitin carboxyl-terminal hydrolase isozyme L5-like; *AGO 1B*, protein argonaute 1B; *ARF8*, auxin response factor 8; *AIP*, auxin-induced protein; *CCR*, cinnamoyl-CoA reductase; *SAMDC*, S-adenosylmethionine decarboxylase; *EIL3*, ethylene-insensitive 3-like 3 protein; *HIR1*, hypersensitive-induced response protein 1; *GK*, glycerol kinase; *PP2C*, protein phosphatase 2C; *MLO*, MLO-like protein; *MYB2*, Myb-related protein Hv33; *GRF8*, growth-regulating factor 8

qRT-PCR analysis of miRNAs and their corresponding target genes will not only verify the accuracy of our degradome sequencing results, but also determine the miRNA-mediated regulatory role of miRNAs and their predicted target genes in sugarcane responses to *S. scitamineum* stress. The results showed that the expression level of 13 predicted target genes in qRT-PCR analysis and degradome sequencing was similar, but not completely consistent (Fig. 6). There was also a certain deviation in the differences of gene expression fold. The predicted target genes and their corresponding miRNAs were expressed in opposite patterns in at least one sugarcane variety. However, the expression patterns of different predicted target genes and their corresponding miRNAs in two sugarcane varieties (YC05–179 and ROC22) varied as follows:

(i) Ubiquitin carboxyl-terminal hydrolase isozyme L5-like (*UCH-L5*), protein argonaute 1B (*AGO 1B*), and *ARF8* followed a negative miRNA-mediated regulatory mode in two sugarcane varieties. The expression patterns of predicted target genes *UCH-L5*, *AGO 1B*, and *ARF8* were opposite to that of their corresponding miRNAs, i.e., *UCH-L5*, *AGO 1B*, and *ARF8* (slightly decreased but not significant) were downregulated, whereas the corresponding miR529-3p, miR168a-5p, and miR160a were upregulated in YC05–179, and the expression pattern in ROC22 was the opposite (Fig. 6A).
(ii) Auxin-induced protein (*AIP*), cinnamoyl-CoA reductase (*CCR*), and S-adenosylmethionine decarboxylase (*SAMDC*) fit the negative miRNA-mediated regulatory mode only in ROC22. While the expression trends of predicted target genes and their corresponding miRNA in YC05–179 were consistent, i.e., *AIP*, *CCR*, and *SAMDC* were upregulated, the corresponding miR5293, nov-mir-132, and miR162a were downregulated in ROC22, whereas these predicted target genes and their corresponding miRNAs were upregulated in YC05–179 (Fig. 6B).
(iii) *EIL3* and *HIR1* were upregulated and followed the negative miRNA-mediated regulatory mode only in ROC22. Whereas the expression level of *EIL3* and *HIR1* in YC05–179 showed little change, the expression amount of their corresponding nov-mir-143 and miR5368 varied greatly and all was upregulated (Fig. 6C).
(iv) Growth-regulating factor 8 (*GRF8*), glycerol kinase (*GK*), *PP2C*, mildew resistance locus o (*MLO*), and Myb-related protein Hv33 (*MYB2*) fit the negative miRNA-mediated regulatory mode only in YC05–179 (Fig. 6D). *GRF8* was downregulated in both YC05–179 and ROC22, and the corresponding nov-mir-18, nov-mir-66, and miR396e-5p were all upregulated in YC05–179, but upregulated in ROC22 only at 5 d. *GK* was upregulated at 2 d and downregulated at 5 d after inoculation with *S. scitamineum* in YC05–179, whereas the corresponding nov-mir-63 showed the opposite pattern. The expression level of *GK* in ROC22 decreased with prolongation of inoculation time. The expression pattern of nov-mir-63 was the same as that in YC05–179. *PP2C* was downregulated and the corresponding nov-mir-97 was upregulated in YC05–179 at 2 d, but both of them were stable in ROC22. *MLO* was downregulated in YC05–179 and ROC22 at 2 d, and both of them recovered at 5 d. Its corresponding nov-mir-10 was significantly upregulated in YC05–179 at 2 d and then declined again at 5 d, whereas the expression pattern in ROC22 was the opposite. The expression level of *MYB2* in YC05–179 was higher than that in ROC22. When the transcript of *MYB2* in YC05–179 was upregulated at 5 d, it remained stable in ROC22, and the corresponding miR858b was upregulated in YC05–179 and peaked at 2 d but remained unchanged in ROC22.

The above results indicated the complexity of miRNA regulation of *S. scitamineum* that infects sugarcane. There was no obvious negative linear regulation between the miRNAs and their predicted target genes, and there were also significant differences in the regulation of expression patterns between different genotypes of sugarcane. The expression level of *AGO 1B*, *UCH-L5*, *AIP*, *CCR*, *EIL3*, *HIR1*, and *SAMDC* significantly changed at 2 d after inoculation, indicating that they responded earlier to *S. scitamineum* infection. *ARF* and *GRF* are associated with plant growth and development [59–61]. The significantly decreased expression level of *GRF8* and the slightly decreased expression level of *ARF8* after inoculation suggests that the infection of *S. scitamineum* might inhibit the growth of sugarcane to a certain extent. MLO is a calcium-binding protein whose expression is negatively correlated with plant disease resistance [62, 63]. The *MLO* gene in sugarcane was downregulated at 2 d after inoculation with *S. scitamineum* compared to ROC22, with a significant decrease in expression level in YC05–179, suggesting that *MLO* responded earlier to smut pathogen infection and that the intensity of the response was greater in the resistant variety than the susceptible one. *MYB* plays a more important role in the defense response of plants to stress [64]. *MYB2* was upregulated in YC05–179 at 5 d after inoculation with *S. scitamineum*, indicating a delay in response. *GK* is a rate-limiting enzyme in the metabolic pathway of glycerol, and its expression level is closely related to the innate immune response in plants [65, 66]. The expression level of *GK* in the resistant variety YC05–179 was increased and higher than that in the susceptible variety ROC22 after infection with *S. scitamineum*, which may corroborate the positive correlation between the expression level of *GK* and smut resistance in sugarcane varieties.

## Discussion

Plant miRNAs mainly regulate target gene expression by mediating target mRNAs cleavage or repressing gene translation during plant development [26, 27]. Degradome sequencing is a high-throughput method to identify miRNAs and their predicted target genes at a certain developmental stage or under specific stress in plants, which in turn may reveal the target genes of miRNAs that are related to plant development or response to stress [28, 29]. This technique has now been successfully applied to various plants such as *Populus tomentosa* [57], cotton [58], rice [67], and peanut [31]. In the present study, the expression of the predicted target genes regulated by miRNA-mediated cleavage in smut-resistant and -susceptible varieties of sugarcane under the infection of *S. scitamineum* was analyzed using degradome sequencing. The results showed that an initial data amount of 122.33 M was obtained on six sugarcane samples. The data amount of each sample was not less than 10 M and the Q30 was > 93%, suggesting that the sequencing quality was relatively high. In addition, the sequence of degraded fragments obtained by degradome sequencing was the same as that of transcriptome sequencing [11], which demonstrates the accuracy of degradome sequencing results.

Degradome sequencing allows the rapid acquisition of miRNA-mediated 3′ cleavage fragments containing 5′ monophosphate groups, and then the identification of target gene degradation sites through depth statistics and comparative analysis on the cleaved fragment [28, 29]. In this study, a total of 309 predicted target mRNAs were detected in six libraries, corresponding to 97 known miRNAs and 112 new miRNAs, as well as 337 degradation sites. Previous studies have shown target mRNAs can be cleaved by miRNAs at multiple degradation sites, resulting in a higher number of degradation sites than target genes [56]. After *S. scitamineum* infection, the negative regulatory role in quantitative expression between partial selected miRNAs and their predicted target genes in two sugarcane genotypes was not extremely high (Fig. 6). It is possible that these potential target genes were regulated by more than one miRNAs at the translational level [68–70].

The target genes were annotated with GO, KEGG, NR, NT, Swiss-Prot and COG databases to obtain their basic information, functional classification, and involved metabolic pathways [71, 72]. In this study, predicted target genes involved in various regulatory processes of life activity such as signal transduction, ion transport, translation and posttranslational modification, energy production and transduction, and metabolism of glycerides. In addition, a miRNA precursor, namely, Sugarcane_Unigene_BMK.40037 (miR171e-3 precursor miRNA), cleaved by nov-mir-219, was found in the YC05–179 at 2 d after inoculation with *S. scitamineum*. The interaction process between sugarcane and *S. scitamineum* is regulated by a multi-gene network system. Correspondingly, the process of sugarcane in response to *S. scitamineum* infection is involved in the regulation of multiple metabolic pathways [73, 74]. The differentially expressed predicted target genes in YC05–179 and ROC22 were basically the same in the classification of targets, mainly playing a catalytic and binding role in response to stimulation, signaling pathway and immune process. KEGG analysis showed that the differentially expressed predicted target genes involved in plant hormone signal transduction, plant-pathogen interactions, oxidative phosphorylation, and other disease-related metabolic pathways, in which plant-pathogen interaction pathways appeared only in the differentially expressed predicted target genes of YC05–179, namely Sugarcane_Unigene_BMK.75694. Sugarcane_Unigene_BMK.75694 is cleaved by nov-mir-63, encodes glycerol kinase, which is involved in energy production and transduction. After inoculating with *S. scitamineum*, the expression of Sugarcane_Unigene_BMK.75694 in YC05–179 was increased with the prolongation of inoculation time, which was opposite to that in ROC22. Sugarcane_Unigene_BMK.62557 (catalase), which participated in the peroxisomal pathway and was regulated by miR858b and nov-mir-88, was only differentially expressed in YC05–179 and ROC22 that were inoculated for 5 d. The expression level of Sugarcane_Unigene_BMK.62557 in YC05–179 was increased with the elongation of inoculation, but was opposite to that in ROC22. This does not agree with our finding that catalase activity was higher at 2 d compared to that at 5 d after inoculation (Fig. 2), which may be because the expression level of other catalase family members had greater changes than that of Sugarcane_Unigene_BMK.62557, thus fitting the trend of changes in the activity of catalase. In addition, YC05–179 and ROC22 also responded to *S. scitamineum* infection through their own differential expression of genes. Therefore, link the changes in transcript levels of predicted target genes to observe biology of infection will provide a better insight on sugarcane in response to smut pathogen attack.

Pathogen invasion triggers various plant immune responses [75, 76]. When infected by pathogens, the responses of plant genes may differ such as early or late stress responses or high or low expression level [11]. miRNAs mainly regulate target gene expression by cleavage, which in turn influences the life processes of plants [26, 27]. At the same time, the response of plants to pathogen infection involves multiple metabolic pathways and several genes [10, 13]. This study showed that inoculation with *S. scitamineum* induces changes in multiple resistance-related metabolic pathways in YC05–179 and ROC22 as following:

### Lignin biosynthesis pathway

Lignin biosynthesis is a branch of the phenylpropanoid metabolic pathway that plays an important role in plant disease resistance [77]. CCR is a key enzyme that catalyzes lignin biosynthesis and promotes the formation of lignin [78, 79]. CCR first catalyzes the formation of corresponding aldehydes by coumaryl-CoA, feruloyl-CoA and sinapoyl-CoA, which are then catalyzed by cinnamyl alcohol dehydrogenase (CAD) to form lignin monomers [78, 79]. Previous studies have found that the reduced expression of *CCR* can cause a significant decrease in lignin content [80]. In addition, *CCR* gene expression in plants is upregulated and the accumulation of lignin monomer is increased during fungal infections [81, 82]. PPO has often been suggested to participate in plant defense against pathogens and pests by promoting the formation of lignin and quinones [56, 83]. Li and Steffens reported that overexpression of *PPO* in tomato plants results in enhanced resistance to *Pseudomonas syringae* pv. *tomato* [56]. In this study, degradome sequencing analysis showed that nov-mir-132 targets the *CCR* gene and regulates its expression by cleavage. After inoculation with *S. scitamineum*, the expression level of *CCR* in YC05–179 and ROC22 was upregulated (Fig. 6B), which coincided with the increase in PPO activity at 2 and 5 d after inoculation (Fig. 2), suggesting that high expression of *CCR* transcript and PPO activity may promote the synthesis and accumulation of lignin to actively respond to *S. scitamineum* infection.

### Ubiquitin-mediated pathway of protein degradation

Ubiquitination is a common protein modification in eukaryotes [84]. Ubiquitin-mediated protein degradation pathways such as jasmonic acid (JA) [85], salicylic acid (SA) [86], and effector-triggered immunity (ETI) [87] are involved in plant immune responses. Deubiquitination enzyme (DUB) has been shown to efficiently cleave ubiquitin-labeled target proteins and regulate ubiquitin-mediated metabolic pathways [88]. UCH-L5, a deubiquitinating enzyme belonging to the ubiquitin carboxy terminal hydrolase family, is closely related to the ubiquitin degradation pathway [89]. In this study, miR529-3p targets *UCH-L5*, was downregulated in YC05–179 after inoculation with *S. scitamineum*, whereas upregulated in ROC22 (Fig. 6A), suggesting that *UCH-L5* may undergo ubiquitin-mediated protein degradation pathways to respond to *S. scitamineum* infection.

### Interaction pathways between plants and pathogens

Interaction between plants and pathogens will stimulate various defense mechanisms in plants such as the formation and accumulation of phytoalexins and disease-related proteins, second messenger production, hypersensitive reactions (HRs), and programmed cell death (PCD). GK catalyzes the phosphorylation of glycerol, which is a key rate-limiting enzyme in the glycerol metabolic pathway [90]. Nonhost resistance 1 (NHO1), a member of GK, is involved in the JA and SA signal transduction pathways and plays a role in plant disease resistance [66, 91]. The *NHO* gene is necessary for the R-gene related pathway and its expression can be activated by flagellin [65]. After inoculation with *Xanthomonas oryzae*, the expression level of *OsNHO1* in rice rapidly increased within 3 h, peaked at 9 h, then gradually decreased and dropped to that of the control at 1 d, indicating that *NHO1* is highly responsive to *X. oryzae* in rice [92]. In this study, nov-mir-63 targets *GK*. KEGG pathway analysis showed that this GK has NHO1 activity and participates in the plant-pathogen interaction pathway and glycerolipid metabolism. Moreover, after inoculation with *S. scitamineum*, the *GK* expression level was upregulated in the resistant variety YC05–179 but downregulated in the susceptible variety ROC22 at 2 d (Fig. 6D), suggesting that the *GK* gene may play a positive role in sugarcane resistance to *S. scitamineum*.

Ca^2+^ is a second messenger involved in plant-pathogen interactions. *MLO* is a recessive susceptible gene. Ca^2+^-mediated *MLO* binds to calmodulin and is negatively correlation to plant disease resistance [62]. Plants often show susceptibility when the *MLO* gene normally expresses the MLO protein, whereas exhibit broad-spectrum disease resistance when the *MLO* gene does not express or express non-functional proteins [63]. Previous studies have shown that *mol* mutations can improve plant resistance to bacteria and fungi [93, 94]. Therefore, in plants, the *MLO* gene is equivalent to a susceptible gene. The *MLO* gene has been associated with resistance to powdery mildew of wheat [95], as well as resistance to leaf blight of barley [96], necrosis of infected parts, and suppression of pathogen expansion [96]. In this study, we found that nov-mir-10 could target *MLO*. After inoculation with *S. scitamineum*, *MLO* was downregulated in YC05–179 at 2 d, and its expression level was lower than that in ROC22 (Fig. 6D). It has been suggested that nov-mir-10 in YC05–179 could more efficiently cleave *MLO*, thereby reducing the inhibition of defense response by the MLO protein and improving sugarcane resistance to smut pathogen.

### Phytohormone signal transduction pathway

Phytohormones play a role in plant growth and development and responses to environmental stresses [97]. Phytohormones mainly include auxin, gibberellin acid (GA), cytokinins (CKs), ethylene (ETH), SA, JA, polyamines, and brassinosteroids (BRs). Among these phytohormones, SA, JA, ETH, and BR are disease resistance signaling molecules, whereas ETH and auxin biosynthesis promote each other and participate in plant responses to stress [97]. Ethylene insensitive 3 (*EIN3*) and EIN3-like 1 (*EIL1*) are transcription factors in the ethylene signaling pathway that promote the expression of ethylene response factor 1 (*ERF1*) and thereby regulate defense genes in response to pathogen infection [98]. In our previous proteomics research on sugarcane at 2 d after inoculation with *S. scitamineum* by iTRAQ analysis, four proteins involved in the ETH pathway were observed, including two 1-aminocyclopropane-1-carboxylate oxidases (ACOs) that were responsible for ETH biosynthesis, as well as one EIN3 and one ERF1 that was responsible for ETH signaling [12]. One ACO (gi35014290) was upregulated in both sugarcane genotypes (YC05–179 and ROC22) and the other one (gi41615358) was downregulated in ROC22 only, but remained unchanged in YC05–179. One EIN3 (Sugarcane_Unigene_BMK.65773) and one ERF1 (gi35045219) were both upregulated in YC05–179, whereas it remained stable in ROC22 [12]. In this study, another *EIL3* gene (Sugarcane_Unigene_BMK.64656) was targeted by nov-mir-143. After inoculation with *S. scitamineum*, the expression of *EIL3* (Sugarcane_Unigene_BMK.64656) in YC05–179 was stable, but in ROC22 it was rapidly increased at 2 d and 5 d (Fig. 6C). It is speculated that smut pathogen attack enhances sugarcane ethylene metabolism, which is involved in *EIL3* in ROC22 and in turn favors resistance to *S. scitamineum* infection [99]. However, YC05–179 responded to smut pathogen infection by other family genes in the ETH pathway. In addition, we also identified an *AIP* gene. KEGG analysis showed that AIP has small auxin up RNAs (SAUR) activity. SAUR plays a negative regulatory role in auxin biosynthesis and transport [100]. After inoculation with *S. scitamineum*, the expression level of *AIP* was upregulated in YC05–179 at 2 and 5 d and increased at 2 d in ROC22 by qRT-PCR (Fig. 6B). However, since this result differs from the *AIP* expression patterns obtained from degradome sequencing, further experiments are needed to verify how *AIP* responds to the infection by *S. scitamineum*. *SAMDC* and S-adenosylmethionine synthetase (*SAMS*) are important genes for polyamine biosynthesis [101, 102]. Previous studies have shown that overexpression of the *SAMDC* gene can increase wilt resistance that is induced by *Verticillium dahliae* and *Fusarium oxysporum* in transgenic tobacco plants [103]. The expression of a *SAMS* gene in sugarcane increases after infection with *S. scitamineum* [104]. We found that miR162a targets *SAMDC* after inoculation with *S. scitamineum* and promotes the expression of *SAMDC* in both YC05–179 and ROC22 at 2 d (Fig. 6B), indicating that infection by *S. scitamineum* may enhance polyamine metabolism pathway to improve sugarcane smut resistance at the early stage.

### Resistance-related transcription factors

Infection by pathogenic bacteria can stimulate plant transcription factors to participate in defensive responses. MYB is a typical transcription factor in plants that regulates phenylpropanoid metabolism [105] and is involved in hormonal signal transduction pathways such as auxin [106], JA [107], and ETH [107]. MYB is also involved in the systematic acquired resistance (SAR) and HR reactions [108]. Previous studies have found that MYB regulates PAL synthesis and is a positive regulator of phenylpropanoid anabolism [105]. In addition, MYB can be induced by exogenous stresses such as JA and SA, or TMV infection, and it can also activate disease-resistant defense responses involving the *PR* gene [109]. In this study, we found that *MYB2* could be targeted by miR858b. After 5 d of inoculation with *S. scitamineum*, *MYB2* gene was upregulated in YC05–179 and remained stable in ROC22 (Fig. 6D), which coincided with the expression pattern of PPO activity (Fig. 2), suggesting that miR858b may have different cleavage effects on *MYB2* then regulate PAL synthesis in different resistant varieties of sugarcane, ultimately leading to different levels of resistance to smut in YC05–179 and ROC22. Similarly, Yang et al. demonstrated that a novel anther-specific *myb* gene (*NtMYBAS1*) from tobacco was a functional anther-specific transcription factor, which was likely to be a positive regulator of PAL synthesis in sporophytic [105].

### miRNA feedback regulation

Previous studies have found that miRNAs can regulate target gene expression and involved in plant growth, development, and stress response, but also feedback regulate their metabolic synthesis [27]. Xie et al. found that *Arabidopsis dicer-like1* (*DCL1*), which plays an important role in the formation of mature miRNAs, is subject to negative feedback regulation through the activity of miR162 [110]. AGO protein is an important part of RNA-induced silencing complex (RISC) with the function of cleaving miRNA target gene or inhibiting translation [111]. miR168 can target AGO protein and thus regulate the miRNA-regulated target genes in plants through changes in AGO protein expression [111]. In this study, *AGO 1B* was targeted by miR168a-5p. After inoculation with *S. scitamineum*, the upregulated expression of miR168a-5p in YC05–179 causes a decrease in the expression level of target *AGO 1B* and may promote miRNA-mediated accumulation of disease-related target genes, which in turn resists further infection of *S. scitamineum*. Meanwhile, after inoculation with *S. scitamineum*, the expression patterns of *AGO 1B* and miR168a-5p in ROC22 were opposite to that in YC05–179, i.e., miR168a-5p was downregulated and the expression level of *AGO 1B* was upregulated, suggesting that the miRNA self-feedback pathway may involve in sugarcane responses to *S. scitamineum*.

## Conclusions

In the present study, the *S. scitamineum* was rapidly proliferated and the enzyme activities of POD, SOD, CAT, PPO, PAL, and TAL in the reactive oxygen species metabolic pathway and phenylpropanoid metabolic pathway were significantly changed at 2 and 5 d in the compatible and incompatible interactions between sugarcane and *S. scitamineum*. Furthermore, 97 known miRNAs and 112 novel miRNAs with 309 predicted target genes were identified by degradome sequencing. GO and KEGG pathway analyses showed that many predicted target genes enriched in regulation and metabolism. qRT-PCR validation demonstrated that there was no obvious negative regulatory relationship between miRNAs and their target genes. This study elucidates the underlying response of sugarcane to *S. scitamineum* infection and provides useful information on the interplay between miRNAs and their predicted targets. In the future, genetic transformations can be done to further our understanding on whether these genes enhance smut resistance in sugarcane.

## Additional files

Additional file 1: **Table S1.** The qRT-PCR primers of the predicted target genes. **Table S2.** The qRT-PCR primers of miRNAs. **Figure S1.** COG function classification of differentially expressed predicted target genes in YC05–179 (DY) and ROC22 (DR) inoculated with *Sporisorium scitamineum* for 2 d and 5 d. YC05–179, smut-resistant genotype; ROC22, smut-susceptible genotype. (DOCX 1350 kb)

Additional file 2: **Table S3.** List of 309 predicted target mRNAs and their function annotations. (XLSX 55 kb)

## Abbreviations

ACOs: 1-aminocyclopropane-1-carboxylate oxidases
AGO 1B: Protein argonaute 1B
AIP: Auxin-induced protein
ARF: Auxin response factor
BRs: Brassinosteroids
CAD: Cinnamyl alcohol dehydrogenase
CAT: Catalase
CCR: Cinnamoyl-CoA reductase
CKs: Cytokinins
CLP1: Chemocyanin-like protein
CMV: Cucumber mosaic virus
DCL1: Dicer-like1
DUB: Deubiquitination enzyme
EIL3: Ethylene-insensitive 3-like 3 protein
EIN3: Ethylene insensitive 3
ERF1: Ethylene response factor 1
ETH: Ethylene
ETI: Effector-triggered immunity
FDR: False discovery rate
FPKM: Fragments per kilobase of transcript per million mapped reads
GA: Gibberellin acid
GAPDH: Glyceraldehyde-3-phosphate dehydrogenase
GK: Glycerol kinase
GO: Gene ontology
GRF8: Growth-regulating factor 8
HRs: Hypersensitive reactions
JA: Jasmonic acid
KEGG: Kyoto encyclopedia of genes and genomes
LHT1: Lysine histidine transporter 1
miRNAs: MicroRNAs
MLO: Mildew resistance locus o
MYB: v-myb avian myeloblastosis viral oncogene homolog
NAM: No apical meristem
NBT: Nitroblue tetrazolium
NHO1: Nonhost resistance 1
PAL: Phenylalanine ammonia lyase
PARE: Parallel analysis of RNA ends
PCD: Programmed cell death
PERK2: Proline-rich receptor-like protein kinase 2
POD: Peroxidase
PP2C: Protein phosphatase 2C
PPO: Polyphenol oxidase
PTGS: Post-transcriptional gene silencing
PVP: Polyvinylpyrrolidone
RISC: RNA-induced silencing complex
SA: Salicylic acid
SAMDC: S-adenosylmethionine decarboxylase
SAMS: S-adenosylmethionine synthetase
SAR: Systematic acquired resistance
SAUR: Small auxin up RNAs
SERK: Somatic embryogenesis receptor kinase
SOD: Superoxide dismutase
SPL: Squamosa promoter-binding-like protein
TAL: Tyrosine ammonia lyase
TAV: Tomato aspermy virus
UBC5: Ubiquitin-conjugating enzyme E2 5
UCH-L5: Ubiquitin carboxyl-terminal hydrolase isozyme L5-like
