# Supplementary material for: A dynamic degradome landscape on miRNAs and their predicted targets in sugarcane caused by Sporisorium scitamineum stress
Source: BMC Genomics. 2019 Jan 18;20:57. doi: 10.1186/s12864-018-5400-8 (PMC6339412; doi:10.1186/s12864-018-5400-8)
Supplement: Supplementary file 1 — Table S1. The qRT-PCR primers of the predicted target genes. Table S2. The qRT-PCR primers of miRNAs. Figure S1. COG function classification of differentially expressed predicted target genes in YC05–179 (DY) and ROC22 (DR) inoculated with Sporisorium scitamineum for 2 d and 5 d. YC05–179, smut-resistant genotype; ROC22, smut-susceptible genotype. (DOCX 1350 kb) [file 12864_2018_5400_MOESM1_ESM.docx]

**A dynamic degradome landscape on miRNAs and their predicted targets in sugarcane caused by *Sporisorium scitamineum* stress**

**Yachun Su^1,2^**

**E-mail:** **[syc2009mail@163.com](mailto:syc2009mail@163.com)**

**Xinhuan Xiao^1^**

**E-mail: xinhuanxiao2013@126.com**

**Hui Ling^1^**

**E-mail: linghuich@163.com**

**Ning Huang^1^**

**E-mail: hning2012@126.com**

**Feng Liu^1^**

**E-mail: 18359162091@163.com**

**Weihua Su^1^**

**E-mail: suweihua2016@126.com**

**Yuye Zhang^1^**

**E-mail: guang_mi@163.com**

**Liping Xu^1^**

**E-mail:** [**xlpmail@126.com**](mailto:xlpmail@126.com)

**Khushi Muhammad^3^**

**E-mail:** [**sikanderkhushi@hotmail.com**](mailto:sikanderkhushi@hotmail.com)

**Youxiong Que^1,2^***

**E-mail:** [**queyouxiong@126.com**](mailto:queyouxiong@126.com)

^1^Key Laboratory of Sugarcane Biology and Genetic Breeding, Ministry of Agriculture, Fujian Agriculture and Forestry University, Fuzhou 350002, China

^2^Key Laboratory of Ministry of Education for Genetics, Breeding and Multiple Utilization of Crops, College of Crop Science, Fujian Agriculture and Forestry University, Fuzhou 350002, China

^3^Department of Genetics, Hazara University, Mansehra 21300, Pakistan

***Correspondence should be addressed to** [queyouxiong@126.com](mailto:queyouxiong@126.com)

**The full postal address of the submitting author Youxiong Que is as follows:** Key Laboratory of Sugarcane Biology and Genetic Breeding, Ministry of Agriculture, Fujian Agriculture and Forestry University, Fuzhou 350002, China

**Table S1.** The qRT-PCR primers of the predicted target genes

| No. | Predicted target gene name | Predicted target gene ID | Forward primer (5’-3’) | Reverse primer (5’-3’) |
| --- | --- | --- | --- | --- |
| 1 | *AGO 1B* | Sugarcane_Unigene_BMK.66779 | GGAGGTAATGTTGGACGAA | GGCTTGATACTGGACATAGG |
| 2 | *AIP* | Sugarcane_Unigene_BMK.40335 | TCACCTTCCCCTTCTTCT | GTACTTCTTGCCCACCAC |
| 3 | *ARF8* | Sugarcane_Unigene_BMK.63027 | ATACAGGGAGCCAGGCAT | AGGTCGGTGCTGATTCTT |
| 4 | *CCR* | Sugarcane_Unigene_BMK.28594 | TGAGTATGCCTTCTTTGTG | AGAACTGGTATTGCTATGC |
| 5 | *EIL3* | Sugarcane_Unigene_BMK.64656 | GTACATGTGGAAGGTGAAC | GGCAAAAGCCATAGAGAAAG |
| 6 | *GK* | Sugarcane_Unigene_BMK.75694 | GTGAATGATGTCCTGAGC | TAACAGTAGCACCACCATC |
| 7 | *GRF8* | Sugarcane_Unigene_BMK.60551 | CACCGTTCAAGAAAGCAT | GATCAGTCACATTAGCAGC |
| 8 | *HIR1* | Sugarcane_Unigene_BMK.51989 | TAGTGGATGGGCTGAGAG | GACGAGGACTTGGATGAG |
| 9 | *MLO* | Sugarcane_Unigene_BMK.62668 | AGCATAAGAAGCAGAAAGC | GAGACTCAGGACTAAAGAAATG |
| 10 | *MYB2* | gi35098237 | CATCATCAGCCTCCACAA | GCACGAGTTCCAGAAGTT |
| 11 | *PP2C* | Sugarcane_Unigene_BMK.51113 | CGCTACTCTCCTTCCCTA | CGAATCGCCAAACCACAC |
| 12 | *SAMDC* | Sugarcane_Unigene_BMK.67816 | CCACTGCTCTGTCTTATGAT | CTGCTCCACCATGTTGTT |
| 13 | *UCH-L5* | Sugarcane_Unigene_BMK.64654 | GTGCTGATGACCTTGATTG | ATCTGCTCCCTTCTCTTC |
| 14 | *GAPDH* | / | CACGGCCACTGGAAGCA | TCCTCAGGGTTCCTGATGCC |

*AGO 1B*, protein argonaute 1B; *AIP*, auxin-induced protein; *ARF8*, auxin response factor 8; *CCR*, cinnamoyl-CoA reductase; *EIL3*, ethylene-insensitive 3-like 3 protein; *GK*, glycerol kinase; *GRF8*, growth-regulating factor 8; *HIR1*, hypersensitive-induced response protein 1; *MLO*, MLO-like protein; *MYB2*, Myb-related protein Hv33; *PP2C*, protein phosphatase 2C; *SAMDC*, S-adenosylmethionine decarboxylase; *UCH-L5*, ubiquitin carboxyl-terminal hydrolase isozyme L5-like; *GAPDH*, glyceraldehyde-3-phosphate dehydrogenase

**Table S2.** The qRT-PCR primers of miRNAs

| No. | miRNA name | Primer name | Sequence |
| --- | --- | --- | --- |
| 1 | miR168a-5p | miRNA sequence | 5’-TCGCTTGGTGCAGATCGGGAC-3’ |
|  |  | RT primer | 5’-GTCGTATCCAGTGCAGGGTCCGAGGTATTCGCACTGGATACGACGTCCCG-3’ |
|  |  | Forward primer | 5’-CGCGGTCGCTTGGTGCAG-3’ |
|  |  | Reverse primer | 5’-GTGCAGGGTCCGAGGTATTC-3’ |
| 2 | miR5293 | miRNA sequence | 5’-GTGGAGAAGAGGAAGGAAGAAGAA-3’ |
|  |  | RT primer | 5’-GTCGTATCCAGTGCAGGGTCCGAGGTATTCGCACTGGATACGACTTCTTC-3’ |
|  |  | Forward primer | 5’-GTCAGGTGGAGAAGAGGAAGGA-3’ |
|  |  | Reverse primer | 5’-GTGCAGGGTCCGAGGTATTC-3’ |
| 3 | miR160a | miRNA sequence | 5’-TGCCTGGCTCCCTGTATGCCA-3’ |
|  |  | RT primer | 5’-GTCGTATCCAGTGCAGGGTCCGAGGTATTCGCACTGGATACGACTGGCAT-3’ |
|  |  | Forward primer | 5’-CTAATGTGCCTGGCTCCCTG-3’ |
|  |  | Reverse primer | 5’-GTGCAGGGTCCGAGGTATTC-3’ |
| 4 | nov-mir-132 | miRNA sequence | 5’-CACATAAAGGGACAAAAAGGGCA-3’ |
|  |  | RT primer | 5’-GTCGTATCCAGTGCAGGGTCCGAGGTATTCGCACTGGATACGACTGCCCT-3’ |
|  |  | Forward primer | 5’-GCACGCACATAAAGGGACA-3’ |
|  |  | Reverse primer | 5’-GTGCAGGGTCCGAGGTATTC-3’ |
| 5 | nov-mir-143 | miRNA sequence | 5’-TGGCGATGAGGAAAGAAATCCGT-3’ |
|  |  | RT primer | 5’-GTCGTATCCAGTGCAGGGTCCGAGGTATTCGCACTGGATACGACACGGAT-3’ |
|  |  | Forward primer | 5’-GACTGGCGATGAGGAAAGA-3’ |
|  |  | Reverse primer | 5’-GTGCAGGGTCCGAGGTATTC-3’ |
| 6 | nov-mir-63 | miRNA sequence | 5’-GCAGCACCATCAAGATTCACA-3’ |
|  |  | RT primer | 5’-GTCGTATCCAGTGCAGGGTCCGAGGTATTCGCACTGGATACGACTGTGAA-3’ |
|  |  | Forward primer | 5’-GACTCGCAGCACCATCAAGA-3’ |
|  |  | Reverse primer | 5’-GTGCAGGGTCCGAGGTATTC-3’ |
| 7 | miR396e-5p | miRNA sequence | 5’-TCCACAGGCTTTCTTGAACTG-3’ |
|  |  | RT primer | 5’-GTCGTATCCAGTGCAGGGTCCGAGGTATTCGCACTGGATACGACCAGTTC-3’ |
|  |  | Forward primer | 5’-CTCGTATCCACAGGCTTTCTT-3’ |
|  |  | Reverse primer | 5’-GTGCAGGGTCCGAGGTATTC-3’ |
| 8 | nov-mir-66 | miRNA sequence | 5’-TCGCAGGTCACACAAGAAGCAC-3’ |
|  |  | RT primer | 5’-GTCGTATCCAGTGCAGGGTCCGAGGTATTCGCACTGGATACGACAAGTTC-3’ |
|  |  | Forward primer | 5’-CCGTCTTCCACAGCTTTCTT-3’ |
|  |  | Reverse primer | 5’-GTGCAGGGTCCGAGGTATTC-3’ |
| 9 | nov-mir-18 | miRNA sequence | 5’-TTCCACAGCTTTCTTGAACTG-3’ |
|  |  | RT primer | 5’-GTCGTATCCAGTGCAGGGTCCGAGGTATTCGCACTGGATACGACCAGTTC-3’ |
|  |  | Forward primer | 5’-CCGTCTTCCACAGCTTTCTT-3’ |
|  |  | Reverse primer | 5’-GTGCAGGGTCCGAGGTATTC-3’ |
| 10 | miR5368 | miRNA sequence | 5’-AGGGACAGTCTCAGGTAGACA-3’ |
|  |  | RT primer | 5’-GTCGTATCCAGTGCAGGGTCCGAGGTATTCGCACTGGATACGACTGTCTA-3’ |
|  |  | Forward primer | 5’-TACGACAGGGACAGTCTCAGG-3’ |
|  |  | Reverse primer | 5’-GTGCAGGGTCCGAGGTATTC-3’ |
| 11 | nov-mir-10 | miRNA sequence | 5’-GCTGAAAGCACTGTTCGCTGGTT-3’ |
|  |  | RT primer | 5’-GTCGTATCCAGTGCAGGGTCCGAGGTATTCGCACTGGATACGACAACCAG-3’ |
|  |  | Forward primer | 5’-CACTCACGCTGAAAGCACTGTT-3’ |
|  |  | Reverse primer | 5’-GTGCAGGGTCCGAGGTATTC-3’ |
| 12 | miR858b | miRNA sequence | 5’-TTCGTTGTCTGTTCGACCTCG-3’ |
|  |  | RT primer | 5’-GTCGTATCCAGTGCAGGGTCCGAGGTATTCGCACTGGATACGACCGAGGT-3’ |
|  |  | Forward primer | 5’-TCAGTCTTCGTTGTCTGTTCG-3’ |
|  |  | Reverse primer | 5’-GTGCAGGGTCCGAGGTATTC-3’ |
| 13 | nov-mir-97 | miRNA sequence | 5’-AACGAGGGACCTATGGATGGA-3’ |
|  |  | RT primer | 5’-GTCGTATCCAGTGCAGGGTCCGAGGTATTCGCACTGGATACGACTCCATC-3’ |
|  |  | Forward primer | 5’-TCACGAACGAGGGACCTATG-3’ |
|  |  | Reverse primer | 5’-GTGCAGGGTCCGAGGTATTC-3’ |
| 14 | miR162a | miRNA sequence | 5’-TCGATAAACCTCTGCATCCAG-3’ |
|  |  | RT primer | 5’- GTCGTATCCAGTGCAGGGTCCGAGGTATTCGCACTGGATACGACCTGGAT-3’ |
|  |  | Forward primer | 5’-GCGCGCTCGATAAACCTCTG-3’ |
|  |  | Reverse primer | 5’-GTGCAGGGTCCGAGGTATTC-3’ |
| 15 | miR529-3p | miRNA sequence | 5’-GCTGTACCCTCTCTCTTCTTC-3’ |
|  |  | RT primer | 5’-GTCGTATCCAGTGCAGGGTCCGAGGTATTCGCACTGGATACGACGAAGAA-3’ |
|  |  | Forward primer | 5’-TCGACTGCTGTACCCTCTCTC-3’ |
|  |  | Reverse primer | 5’-GTGCAGGGTCCGAGGTATTC-3’ |


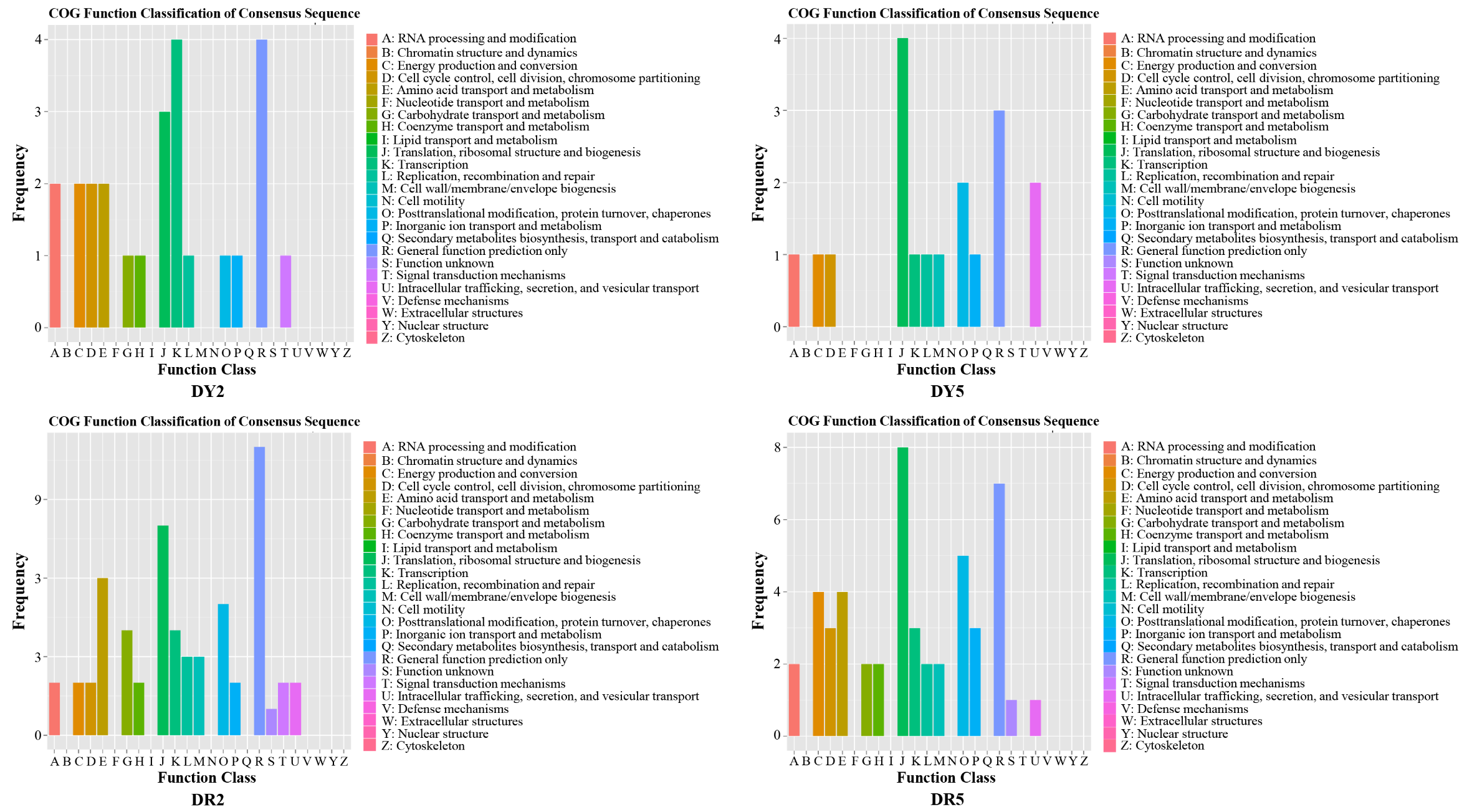


**Figure S1.** COG function classification of differentially expressed predicted target genes in YC05-179 (DY) and ROC22 (DR) inoculated with *Sporisorium scitamineum* for 2 d and 5 d. YC05-179, smut-resistant genotype; ROC22, smut-susceptible genotype
